# Cyst growth in ADPKD is prevented by pharmacological and genetic inhibition of TMEM16A in vivo

**DOI:** 10.1038/s41467-020-18104-5

**Published:** 2020-08-28

**Authors:** Ines Cabrita, Andre Kraus, Julia Katharina Scholz, Kathrin Skoczynski, Rainer Schreiber, Karl Kunzelmann, Björn Buchholz

**Affiliations:** 1 Institut für Physiologie, Universität Regensburg, Universitätsstraße 31, D-93053, Regensburg, Germany; 2 Department of Nephrology and Hypertension, Friedrich-Alexander University Erlangen-Nürnberg, Erlangen, Germany

**Keywords:** Ion channel signalling, Polycystic kidney disease, Nephrons

## Abstract

In autosomal dominant polycystic kidney disease (ADPKD) multiple bilateral renal cysts gradually enlarge, leading to a decline in renal function. Transepithelial chloride secretion through cystic fibrosis transmembrane conductance regulator (CFTR) and TMEM16A (*anoctamin 1*) are known to drive cyst enlargement. Here we demonstrate that loss of *Pkd1* increased expression of TMEM16A and CFTR and Cl^−^ secretion in murine kidneys, with TMEM16A essentially contributing to cyst growth. Upregulated TMEM16A enhanced intracellular Ca^2+^ signaling and proliferation of *Pkd1*-deficient renal epithelial cells. In contrast, increase in Ca^2+^ signaling, cell proliferation and CFTR expression was not observed in *Pkd1/Tmem16a* double knockout mice. Knockout of *Tmem16a* or inhibition of TMEM16A in vivo by the FDA-approved drugs niclosamide and benzbromarone, as well as the TMEM16A-specific inhibitor Ani9 largely reduced cyst enlargement and abnormal cyst cell proliferation. The present data establish a therapeutic concept for the treatment of ADPKD.

## Abstract (web summary)

Polycystic kidney disease (PKD) is characterized by the formation of large renal cysts, which lead to a decline in renal function. Here the authors show that genetic and chemical inhibition of TMEM16A largely reduces cyst enlargement in an in vivo model of autosomal dominant PKD.

## Introduction

Polycystic kidney diseases (PKDs) comprise a number of inherited disorders that lead to bilateral renal cyst development. The most common form, autosomal dominant PKD (ADPKD), affects about 1 in 1000, and accounts for 5–10% of end-stage renal disease. ADPKD is characterized by continuous cyst enlargement over time, leading to compression of adjacent healthy parenchyma^1^. Disease causing mutations in Polycystin-1 (*PKD1*; ~85% of cases) and Polycystin-2 (*PKD2*; ~15% of cases) are known, but the complex mechanisms for cyst development and cyst growth are still poorly understood^2–4^. Cell proliferation and fluid secretion are two essential characteristics of cyst development and enlargement. Epithelial fluid secretion occurs by the chloride channel cystic fibrosis transmembrane conductance regulator (CFTR)^5^. CFTR was also shown to cause expansion of renal cysts^6^. However, the Ca^2+^ activated Cl^−^ channel TMEM16A is known to be essential for fluid secretion into renal cysts in vitro^7–9^. Cyst enlargement is promoted by purinergic signaling and lipid peroxidation, which both activates TMEM16A^10,11^.

Here, we use in vivo and in vitro models for ADPKD and unmask upregulation of expression of TMEM16A and CFTR in kidneys of *Pkd1*-knockout mice and *Pkd1*-knockout MDCK cells. Increased expression of TMEM16A causes enhanced intracellular Ca^2+^ signaling and induces cyst growth, both being normalized by additional knockdown of *Tmem16a*. The results identify upregulated TMEM16A as an essential player in ADPKD. Two FDA-approved drugs which inhibit TMEM16A, niclosamide and benzbromarone^12,13^, and the TMEM16A-specific small molecule Ani9^14^ strongly suppress the cystic phenotype in vivo. The results demonstrate TMEM16A as a central pharmacological target to suppress cyst growth in ADPKD.

## Results

### TMEM16A supports cyst growth in Pkd1-deficient plMDCK cells

**Fig. 1:**
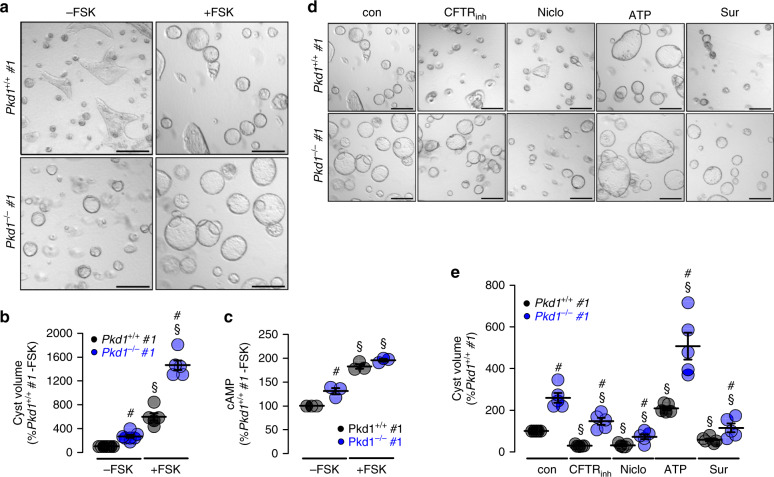
Cyst formation by Polycystin-1-deficient cells depends on cAMP- and Ca^2+^-activated Cl^−^ secretion. **a**, **b** Wild type (*Pkd1*^+*/+*^) and polycystin-1-deficient (*Pkd1*^*−/−*^) principal-like MDCK cells (clones #1) were cultured in a collagen matrix in the absence (−FSK) or presence (−FSK) of 10 µM forskolin. Forskolin induced cyst formation by *PKD1*^*+/+*^ cells within 5 days (^§^*P* < 0.0001). *Pkd1*^*−/−*^ cells demonstrated cyst formation even in the absence of FSK (^#^*P* < 0.0001) and formed larger cysts in the presence of FSK (^#^*P* < 0.0001) (*n* = 105 cysts examined in *n* = 3 independent experiments). **c** Deletion of Polycystin-1 in *Pkd1*^*−/−*^ cells induced an increase in basal intracellular cAMP concentrations (−FSK, ^#^*P* = 0.041). FSK-stimulation further enhanced cAMP levels in both *Pkd1*^*+/+*^ and *Pkd1*^*−/−*^ cells (^§^*P* = 0.0008) (each *n* = 3 independent experiments). cAMP levels in *Pkd1*^*+/+*^ cells were set to 100%). **d**, **e** Cyst growth was strongly inhibited in both *Pkd1*^*+/+*^ and *Pkd1*^*−/−*^ cells by CFTRinh172 (CFTRinh; 10 µM, ^§^*P* < 0.0001), niclosamide (Niclo; 1 µM, ^§^*P* = 0.0001), and suramin (Sur; 100 µM, ^§^*P* = 0.002), but was further augmented by ATP (10 µM, ^§^*P* = 0.002). Bars 200 μm (*n* = 87 cysts examined in *n* = 3 independent experiments). Mean and error bars indicating ±SEM. ^#^Unpaired two-sided *t* test comparing *Pkd1*^*+/+*^ *with Pkd1*^*−/−*^, ^§^unpaired two-sided *t* test comparing effects by forskolin, ATP or inhibitors. Source data are provided as a Source Data file.

In ADPKD, cysts mainly originate from principal cells of the collecting duct^9,15^. We have established an in vitro cyst model by means of a subclone of collecting duct MDCK cells which highly resembles principal cells (principal-like (pl)MDCK)^9^. We previously demonstrated that blocking of TMEM16A inhibits plMDCK cyst growth in a collagen matrix^8,11^. This is now investigated by eliminating expression of *Pkd1* in two plMDCK cell clones (*Pkd1*^−*/−*^; clones #1 and #2) using CRISPR/Cas9 (Supplementary Fig. 1). *Pkd1*-knockout induces upregulation of expression of the ion channels TMEM16A, CFTR, and the purinergic receptor P2Y2 (Supplementary Fig. 2). Knockdown of *Pkd1* (*Pkd1*^−*/−*^) causes spontaneous cyst growth, which is much less pronounced in control cells (*Pkd1*^*+/+*^). Additional stimulation with forskolin further augments cyst size which is more distinct in *Pkd1*^*−/−*^ than in *Pkd1*^*+/+*^ cysts (Fig. 1a, b). Intracellular cAMP levels are enhanced in *Pkd1*^*−/−*^ cells under control condition and are further enhanced after stimulation with forskolin (Fig. 1c). Forskolin, i.e., cAMP does not only increase cyst size, but also induces cyst formation. *Pkd1*^*+/+*^ cells in the absence of forskolin occasionally show tubule-like structures (Fig. 1a, left upper panel; Supplementary Fig. 3a, left upper panel) which are completely absent in *Pkd1*^*−/−*^ cells (Fig. 1a, right, upper panel; Supplementary Fig. 3a, right upper panel). Tubular structures are also absent in *Pkd1*^*+/+*^ cells when treated with forskolin. These observations suggest that increase in basal cAMP upon deletion of *Pkd1* drives cells into cyst formation.

Cyst growth in the presence of forskolin is attenuated by inhibition of CFTR using CFTRinh172. Inhibition is even more pronounced in the presence of the TMEM16-inhibitor niclosamide. Cyst growth is also inhibited by blocking purinergic receptors with suramin, but, in contrast, is further increased in the presence of ATP (Fig. 1d, e)^10^. The data obtained for clone #1 are reproduced in a second clone #2 (Supplementary Fig. 3). This suggests upregulation of CFTR- and TMEM16A-dependent Cl^−^ secretion in the absence of *PKD1*, which drives the cystic phenotype.

### Renal tubule-specific knockout of Pkd1 upregulates expression of TMEM16A and drives cyst growth

**Fig. 2:**
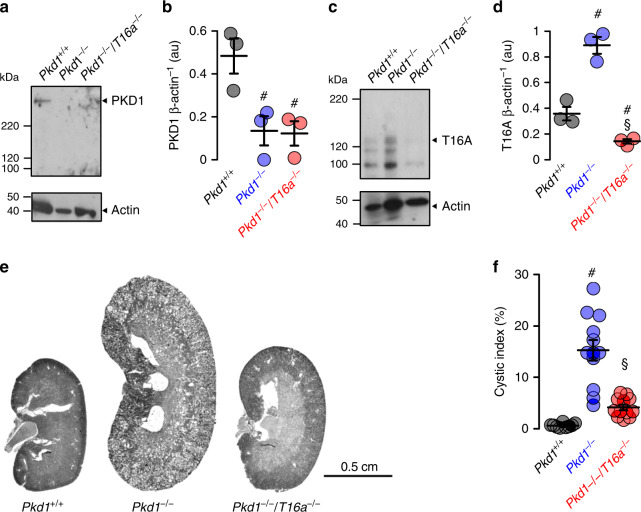
Tubule-specific deletion of TMEM16a reduces cyst progression in an ADPKD mouse model. **a**–**d** Western blotting from whole kidney lysates detecting tubule-specific deletion of *Pkd1* (450 kDa) (^#^*P* = 0.03) and increase in TMEM16A expression (glycosylated 130 kDa and non-glycosylated forms^34^) (^#^*P* = 0.003) (10 weeks after induction at postnatal days 20–22 using tamoxifen. Mice co-deleted for *Pkd1* and *Tmem16a* (*Pkd1*^*−/−*^/*T16a*^*−/−*^) showed reduced *Pkd1* expression (^#^*P* = 0.022) and TMEM16A expression (^§^*P* = 0.0004) (*n* = 3 animals each). **e**, **f** Tubule-specific knockout of *Pkd1* (*Pkd1*^*−/−*^) induced polycystic kidney disease (^#^*P* < 0.0001, *n* = 11 animals), which was largely reduced in mice with an additional knockout of *Tmem16a* (*Pkd1*^*−/−*^/*T16a*^*−/−*^) (^§^*P* = 0.0001, *n* = 11 animals). Non-induced *Pkd1*^fl;fl^ mice served as control (*Pkd1*^*+/+*^) (*n* = 8 animals). Corresponding cystic indices (defined as cortical cyst area normalized to whole cortex area). Mean and error bars indicating ±SEM. ^#^Unpaired two-sided *t* test comparing *Pkd1*^*+/+*^ *with Pkd1*^*−/−*^, ^§^unpaired two-sided *t* test comparing *Pkd1*^*−/−*^ with *Pkd1*^*−/−*^/*T16a*^*−/−*^. Source data are provided as a Source Data file.

We use Ksp*CreER*^T2^;*Pkd1*^lox;lox^ (*Pkd1*^*−/−*^) mice to obtain tamoxifen inducible tubule-specific *Pkd1* knockout^16,17^. Tamoxifen is applied for 3 days starting at postnatal day 20. Kidneys isolated 10 weeks after deletion of *Pkd1* demonstrates clear knockdown of *Pkd1*-expression as shown by Western blotting and when compared to non-induced mice serving as controls (*Pkd1*^*+/+*^) (Fig. 2a, b). In contrast, expression of TMEM16A is upregulated in *Pkd1*^*−/−*^ kidneys (Fig. 2c, d). Knockdown of *Pkd1* induces PKD with multiple renal cysts and enlarges kidneys. The calculated cystic index (c.f. “Methods”) is strongly enhanced (Fig. 2e, f). In contrast, additional knockout of *Tmem16a* in *Pkd1*^*−/−*^ mice (*KspCreER*^*T2*^*;Pkd1*^lox;lox^:*T16a*^lox;lox^), hereafter termed *Pkd1*^*−/−*^/*T16a*^−/−^ (Fig. 2a–d), results in a significantly attenuated cystic phenotype (Fig. 2e, f). The results demonstrate an essential role of TMEM16A for proliferation of cysts, electrolyte secretion into the cysts, or both^8,18^ (Fig. 2e, f).

### Knockdown of Pkd1 causes upregulation of tubular expression of CFTR and TMEM16A

**Fig. 3:**
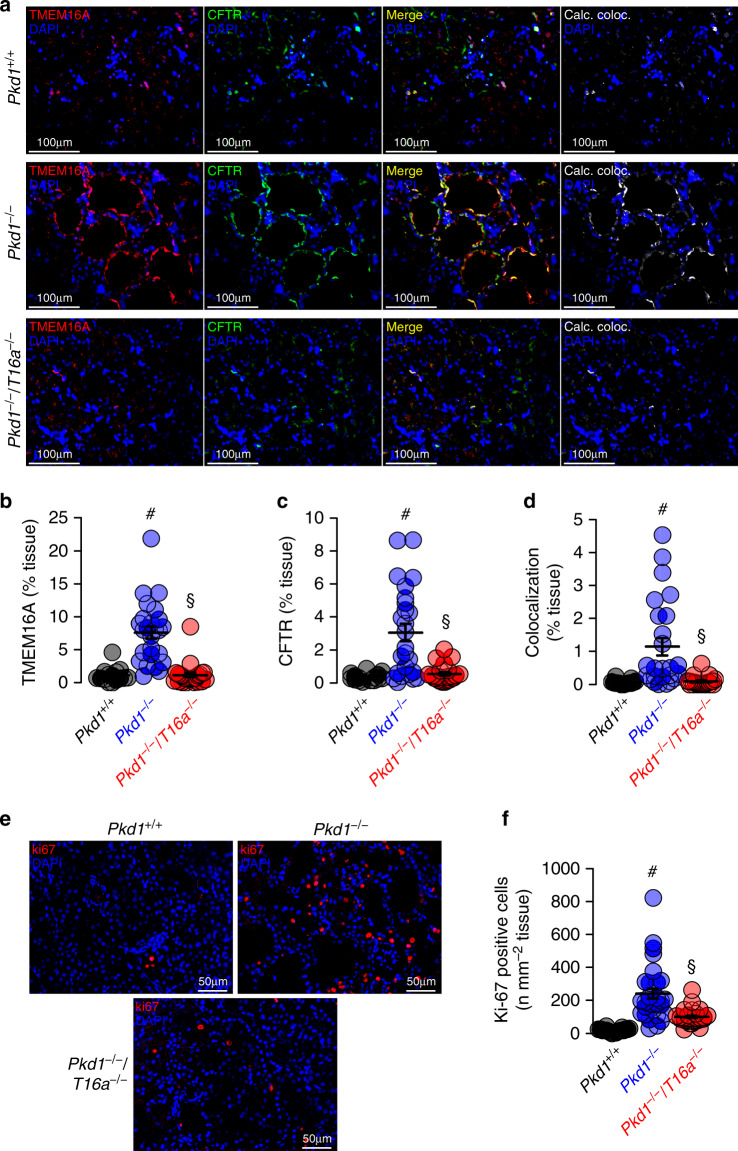
Expression of CFTR and cell proliferation in Pkd1−/− kidneys depends on TMEM16A. **a** Enhanced tubular epithelial expression of CFTR and TMEM16A in kidneys from *Pkd1*^*−/−*^ mice that was abolished in kidneys from *Pkd1*^*−/−*^/*T16a*^*−/−*^ double-knockout mice. Both, CFTR and TMEM16A were colocalized in the apical membrane of the cyst epithelium. (blue; DAPI). **b**–**d** Expression of TMEM16A (^#^*P* < 0.0001) and CFTR (^#^*P* < 0.0001) were enhanced (*n* = 25 independent images over 5 animals) in *Pkd1*^*−/−*^ mice. Significant colocalization of TMEM16A and CFTR was detected (^#^*P* = 0.0006) using colocalization finder algorithm (ImageJ, V.1.49 by Laummonerie and Mutterer). Expression of CFTR (^§^*P* = 0.0006) and TMEM16A (^§^*P* = 0.0006), as well as colocalization (^§^*P* = 0.0001) were lower in *Pkd1*^*−/−*^/*T16a*^*−/−*^ mice. **e**, **f** ki-67 staining was enhanced in tubular epithelial cells from *Pkd1*^*+/+*^ (^#^*P* < 0.0001) animals but was lower in cells from *Pkd1*^*−/−*^/*T16a*^*−/−*^ kidneys (^§^*P* < 0.0001) (*n* = 25 independent images from 5 animals). Mean and error bars indicating ±SEM. ^#§^one-way ANOVA and Tukey’s post-hoc test comparing *Pkd1*^*+/+*^ with *Pkd1*^*−/−*^ and *Pkd1*^*−/−*^ with *Pkd1*^*−/−*^/*T16a*^*−/−*^, respectively. Source data are provided as a Source Data file.

Induction of cyst formation by downregulation of *Pkd1* (*Pkd1*^*−/−*^) is paralleled by upregulation of TMEM16A expression (Fig. 2c). Immunolabelling of TMEM16A and CFTR indicate upregulation of both ion channels in the cyst epithelium of *Pkd1*^*−/−*^ kidneys, where both ion channels are strongly colocalized (Fig. 3a–c, Supplementary Figs. 4 and 5a). In contrast to cystic kidneys, TMEM16A is much less abundant in normal kidneys, where it is found to be expressed in apical membranes and in the primary cilium^19^ (Supplementary Fig. 5b, c). Remarkably, enhanced expression of CFTR in cystic kidneys is entirely reversible upon additional knockout of *Tmem16a* in *Pkd1*^*−/−*^/*T16a*^*−/−*^ animals (Fig. 3a–d and Supplementary Fig. 4). These results confirm the relationship between CFTR and TMEM16A detected earlier, demonstrating the requirement of TMEM16A for proper expression and function of CFTR in mouse and human airway and intestinal epithelial tissues^7,20^. Earlier studies showed a strong correlation between expression of TMEM16A and cell proliferation (reviewed in Refs. ^18,21^). This is also observed in the present study using the proliferation marker ki-67. Proliferation is enhanced in *Pkd1*^*−/−*^ kidneys, but is lowered to almost normal levels in *Pkd1*^*−/−*^/*T16a*^*−/−*^ double-knockout kidneys (Fig. 3e, f).

**Fig. 4:**
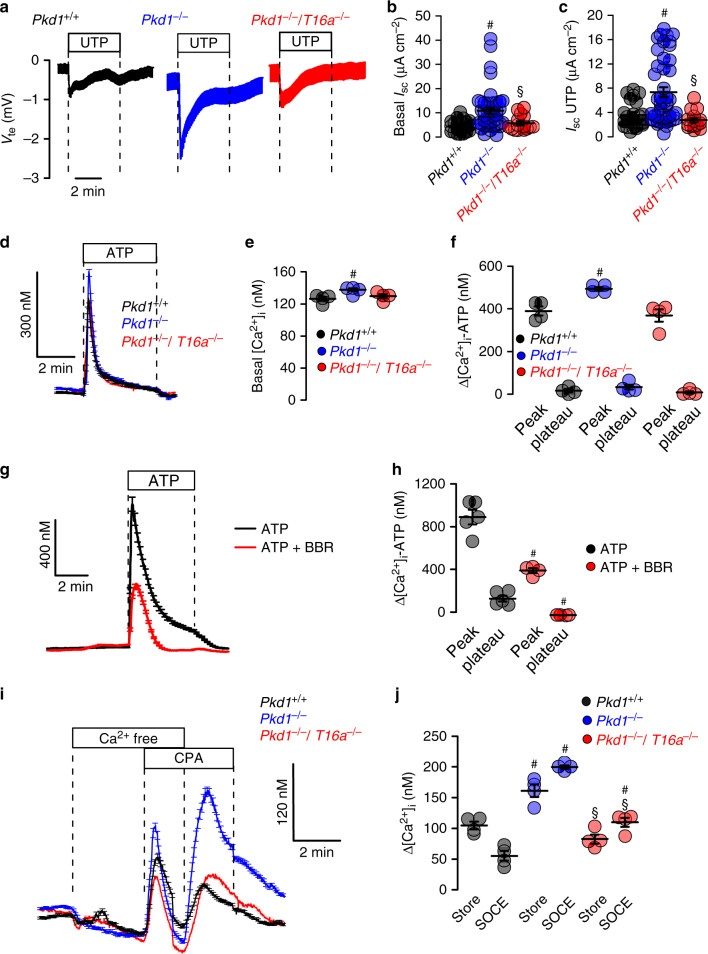
Cl^−^ secretion and enhanced Ca^2+^ signaling by knockdown of Polycystin-1 in primary renal epithelial cells. **a** Original Ussing chamber recordings from polarized grown primary medullary epithelial cells from *Pkd1*^*+/+*^, *Pkd1*^*−/−*^, and *Pkd1*^*−/−*^/*T16a*^*−/−*^ kidneys. **b**, **c** Summaries for calculated basal and UTP (100 µM) activated short-circuit currents (*I*_sc_) demonstrating enhanced basal (^#^*P* = 0.0007) and UTP-activated (^#^*P* = 0.0028) Cl^−^ secretion in epithelia lacking expression of Polycystin-1 (*n* = 45 independent monolayers from 5 animals each). Enhanced basal and UTP-activated Cl^−^ secretion was abolished in *Pkd1*^*−/−*^/*T16a*^*−/−*^ cells (^§^*P* < 0.024 and ^§^*P* < 0.001, respectively). **d**–**f** Basal and ATP (100 µM) induced Ca^2+^ increase were larger (^#^*P* = 0.038 and ^#^*P* = 0.019, respectively) in primary epithelial cells from *Pkd1*^*−/−*^ but from *Pkd1*^*−/−*^/*T16a*^*−/−*^ kidneys (*n* = 44 cells from 4 independent cultures of 4 animals each). **g**, **h** Original recording and summary demonstrating the inhibitory effect of benzbromarone (BBR, 10 µM) on ATP (100 µM) induced Ca^2+^ peak (^#^*P* < 0.0001) and plateau (^#^*P* = 0.005) (*n* = 129 cells from 5 independent cultures of 5 animals each). **i, j** Original recordings and summaries of cyclopiazonic acid (CPA, 10 µM) induced ER Ca^2+^ store release (Store) and activation of store-operated Ca^2+^ influx (SOCE) in medullary primary epithelial cells. Store release (^#^*P* = 0.005) and SOCE (^#^*P* < 0.0001) were larger in *Pkd1*^*−/−*^ cells, but were reduced in cells from *Pkd1*^*−/−*^/*T16*^*−/−*^ kidneys (^§^*P* < 0.0001 and ^§^*P* < 0.0001, respectively) (*n* = 117 cells from 4 independent cultures of 5 animals each). Mean and error bars indicating ±SEM. ^#§^One-way ANOVA and Tukey’s post-hoc test comparing *Pkd1*^*+/+*^ with *Pkd1*^*−/−*^ and *Pkd1*^*−/−*^ with *Pkd1*^*−/−*^/*T16a*^*−/−*^, respectively. Source data are provided as a Source Data file.

Medullary epithelial cells isolated from *Pkd1*^*+/+*^, *Pkd1*^*−/−*^, and *Pkd1*^*−/−*^/*T16a*^*−/−*^ mice are grown on permeable supports to analyze Ca^2+^ dependent ion transport by TMEM16A. Transepithelial voltage deflections are induced by purinergic stimulation with UTP, and both basal and UTP-activated short-circuit currents are enhanced in renal epithelium lacking expression of *Pkd1* (Fig. 4a–c). In contrast, renal epithelium from mice with double knockout of *Pkd1* and Tmem16a demonstrate normal ion transport properties, indicating the role of TMEM16A for enhanced ion transport in ADPKD (Fig. 4a–c and Supplementary Fig. 6a–c). Enhanced Ca^2+^ dependent Cl^−^ secretion in *Pkd1*^*−/−*^ cells is also well detected in whole cell patch-clamp recordings as well as iodide quenching experiments (Supplementary Fig. 6d–g). Importantly, Cl^−^ secretion activated by increase in intracellular cAMP (IBMX and forskolin; IF) is also enhanced in *Pkd1*^*−/−*^ cells, but not in epithelial cells from *Pkd1*^*−/−*^/*T16a*^*−/−*^-double knockout animals (Supplementary Fig. 6h, i).

### Enhanced ion secretion in Pkd1^−/−^ animals is due to upregulated Ca^2+^ signaling

**Fig. 5:**
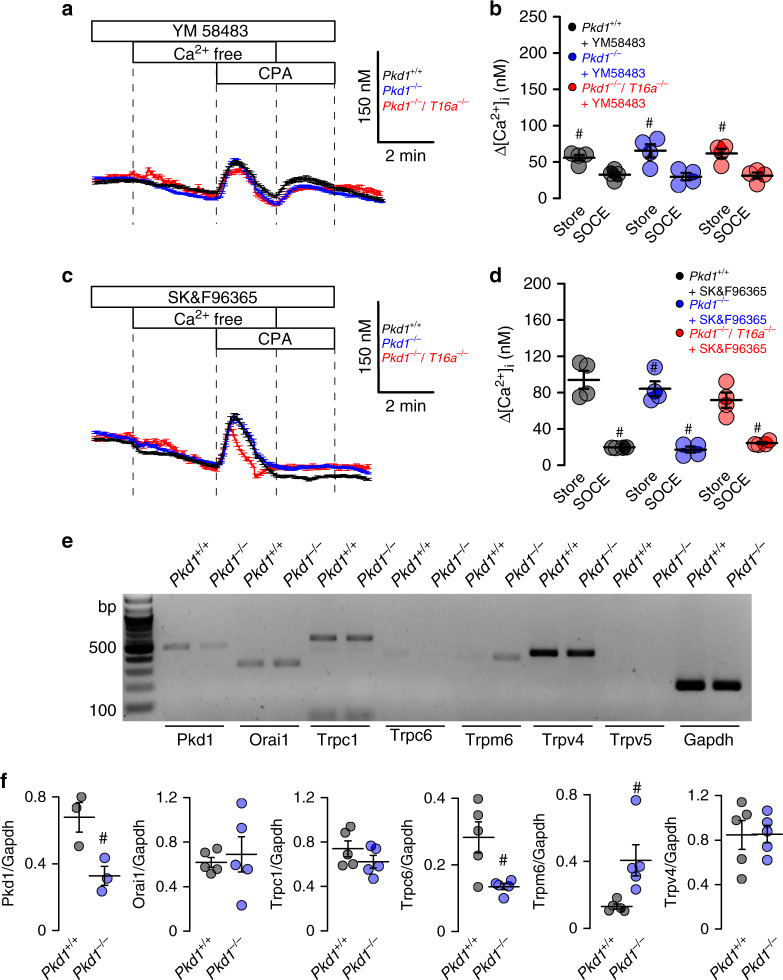
Inhibition of enhanced store-operated Ca^2+^ entry (SOCE) by TRP and Orai channel inhibitors. **a**–**d** Original recordings and summaries showing inhibition of upregulated ER Ca^2+^ store release (Store) and store-operated Ca^2+^ entry (SOCE) in primary renal epithelial cells from *Pkd1*^*−/−*^ mice in the presence of 5 µM Orai channel inhibitor YM58483 (^#^*P* < 0.0001 and ^#^*P* < 0.0001, respectively) and 5 µM TRP-channel inhibitor SK&F96365 (^#^*P* < 0.0001 and ^#^*P* < 0.0001, respectively) (*n* = 73 cells from 5 independent cultures of 4 animals each). **e**, **f** Semiquantitative RT-PCR analysis of Orai1 and TRP-channel (Trpc1, Trpc6, Trpm6, Trpv4, Trpv5) expression in *Pkd1*^*+/+*^ and *Pkd1*^*−/−*^ renal epithelial cells. In *Pkd1*^*−/−*^ cells mRNA for Pkd1 (^#^*P* = 0.03) and Trpc6 (^#^*P* = 0.03) were reduced, while mRNA for Trpm1 was enhanced (^#^*P* = 0.03) (*n* = 5 reactions from 3 animals each). Mean and error bars indicating ±SEM. ^#§^One-way ANOVA and Tukey’s post-hoc test comparing *Pkd1*^*+/+*^ with *Pkd1*^*−/−*^ and *Pkd1*^*−/−*^ with *Pkd1*^*−/−*^/*T16a*^*−/−*^, respectively. Source data are provided as a Source Data file.

Enhanced intracellular Ca^2+^ signaling is observed in cells expressing TMEM16A^22–24^. We therefore compare intracellular Ca^2+^ signals in primary medullary epithelial cells isolated from *Pkd1*^*−/−*^ animals, *Pkd1*^*−/−*^/*T16a*^*−/−*^ double knockout and *Pkd1*^*+/+*^ animals. Basal Ca^2+^ levels and ATP-induced Ca^2+^ rise are both enhanced in *Pkd1*^*−/−*^ cells when compared to double knockout and wt cells (Fig. 4d–f). ATP-induced Ca^2+^ increase is strongly inhibited by the TMEM16A-inhibitor benzbromarone^13^, supporting the role of TMEM16A for intracellular Ca^2+^ signaling (Fig. 4g, h). We employ a Ca^2+^ store release protocol by removing extracellular Ca^2+^ and emptying the endoplasmic reticulum (ER) Ca^2+^ store using the ER Ca^2+^ pump inhibitor cyclopiazonic acid (CPA). We find that deletion of *Pkd1* enhances Ca^2+^ release and largely enhances store-operated Ca^2+^ entry (SOCE) after re-adding extracellular Ca^2+^ (Fig. 4i, j). Thus, Ca^2+^ release and SOCE are coupled and are augmented by deletion of *Pkd1*, probably due to upregulation of TMEM16A^23,25–27^. SOCE is potently blocked by the inhibitors of Orai channels and transient receptor potential (Trp) channels, YM58483 and SK&F96365, respectively, suggesting a contribution of both channels to enhanced SOCE in *Pkd1*^*−/−*^ cells (Fig. 5a–c). Analysis of expression of renal epithelial Trp channels suggests upregulation of Trpm6, which, however, appears unlikely to be in charge of enhanced Ca^2+^ entry as Trpm6 is not inhibited by SK&F96365^28^ (Fig. 5e, f). ER tethering by TMEM16A is therefore likely to be cause for enhanced Ca^2+^ signaling^22,23^.

### FDA-approved inhibitors of TMEM16A and Ani9 attenuate renal cyst growth in vivo

**Fig. 6:**
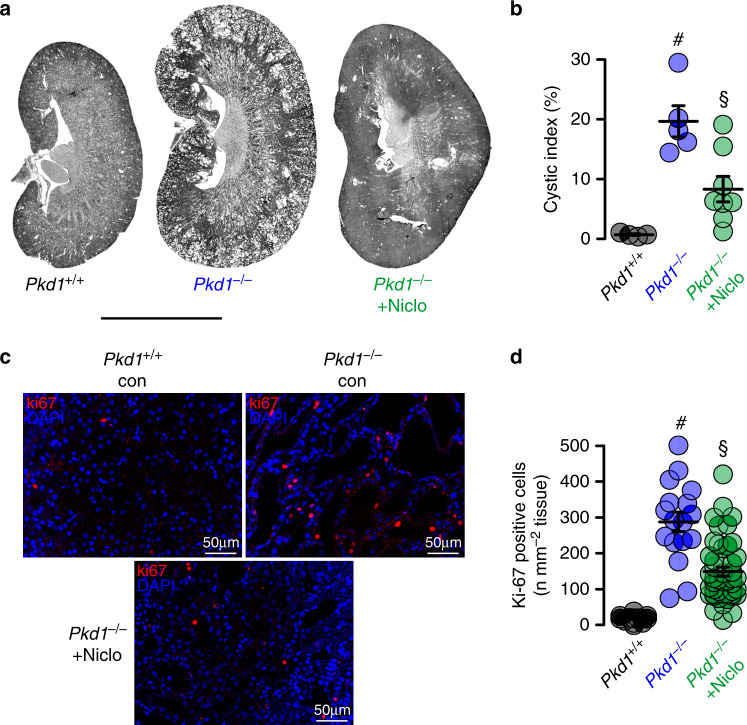
Niclosamide inhibits polycystic kidney disease in a mouse model for ADPKD. **a**, **b** Representative images of kidneys from non-induced (*Pkd1*^*+/+*^) (*n* = 4 animals) and induced (*Pkd1*^*−/−*^) mice, untreated (*n* = 5 animals) or treated with niclosamide (*n* = 8 animals). Scale bar 5000 µm. Tubule-specific deletion of Pkd1 (*Pkd1*^*−/−*^) was induced at postnatal days 20–22. Thereafter, mice were either treated with standard diet or diet supplemented with 0.2% niclosamide (Niclo) for 10 weeks. *Pkd1*^*+/+*^ mice fed with standard diet served as control. Cyst formation in *Pkd1*^*−/−*^ (^#^*P* = 0.003) was strongly inhibited by niclosamide treatment (^§^*P* = 0.005). **c**, **d** Proliferating cells identified in *Pkd1*^*+/+*^ and *Pkd1*^*−/−*^ kidneys by the proliferation marker ki-67 (red). Proliferation was enhanced in *Pkd1*^*−/−*^ kidneys (^#^*P* < 0.0001), but was significantly inhibited by niclosamide (^§^*P* < 0.0001). Number of ki-67 positive cells was normalized to the tissue area (*n* = 25 independent images from 5 animals each). Mean and error bars indicating ±SEM. ^#§^One-way ANOVA and Tukey’s post-hoc test. Source data are provided as a Source Data file.

The present data indicate an essential contribution of TMEM16A to the development of PKD. Recent studies identified two FDA-approved drugs, niclosamide and benzbromarone, as potent inhibitors of TMEM16A^12,13^. We therefore examined if treatment with niclosamide and benzbromarone in vivo will inhibit enlargement of renal cysts in *Pkd1*^*−/−*^ animals. To that end, *Pkd1*^*−/−*^ animals are maintained at normal diet or are fed a diet supplemented with 0.2% niclosamide, which is well tolerated. Animals are sacrificed 10 weeks later and kidneys are analyzed. The animals are monitored daily. No side effects are observed, but in contrast, normalization of weight gain is observed. Cyst development is strongly attenuated in niclosamide-treated mice when compared to animals on standard diet (Fig. 6a, b). Proliferative activity is analyzed by staining medullary epithelial cells with the proliferation marker Ki-67. Knockdown of *Pkd1* largely enhances proliferation, which is significantly reduced by treatment with niclosamide (Fig. 6c, d).

**Fig. 7:**
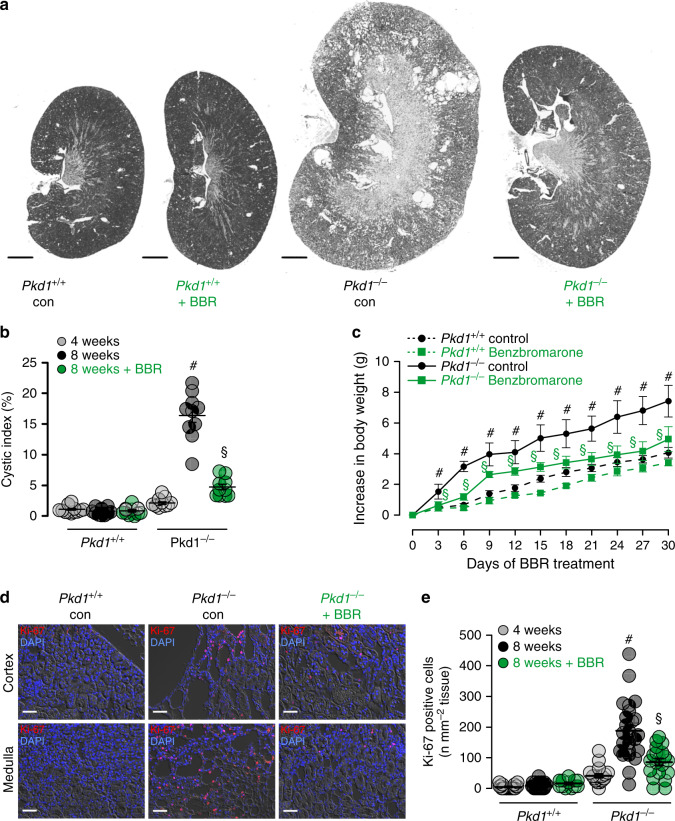
Benzbromarone inhibits polycystic kidney disease in a mouse model for ADPKD. **a** Representative images of kidneys from non-induced (*Pkd1*^*+/+*^) and induced (*Pkd1*^*−/−*^) mice (*n* = 5 animals each), untreated (daily i.p. injection of corn oil) or treated with benzbromarone in corn oil (BBR, 1 mg/kg/day, i.p.) for 30 days. Scale bars: 1000 µm. **b** Cystic index (%) 8 weeks after induction of *Pkd1*^*+/+*^ and *Pkd1*^*−/−*^ mice, untreated or treated with benzbromarone. Cyst growth was induced in *Pkd1*^*−/−*^ mice (^#^*P* < 0.0001), but was inhibited by benzbromarone (^§^*P* < 0.0001) (*n* = 10 kidneys analyzed from *n* = 5 animals each). **c** Time course of body weight for untreated and benzbromarone-treated *Pkd1*^*+/+*^ and *Pkd1*^*−/−*^ mice. Body weight gain was enhanced in *Pkd1*^*−/−*^ mice (^#^*P* < 0.05), but was inhibited by benzbromarone (^§^*P* < 0.05) (*n* = 8 kidneys analyzed from *n* = 5 animals each). **d**, **e** Proliferating cells identified in *Pkd1*^*+/+*^ and *Pkd1*^*−/−*^ kidneys by the proliferation marker ki-67 (red). Proliferation was upregulated in *Pkd1*^*−/−*^ kidneys (^#^*P* < 0.0001), but was inhibited by benzbromarone (^§^*P* < 0.0001) (25 independent images from *n* = 5 animals each). Scale bar 50 µm. Number of ki-67 positive cells was normalized to the tissue area. Mean and error bars indicating ±SEM. ^#§^One-way ANOVA and Tukey’s post-hoc test comparing *Pkd1*^*+/+*^ with *Pkd1*^*−/−*^ and *Pkd1*^*−/−*^ with *Pkd1*^*−/−*^/*T16a*^*−/−*^, respectively. Source data are provided as a Source Data file.

**Fig. 8:**
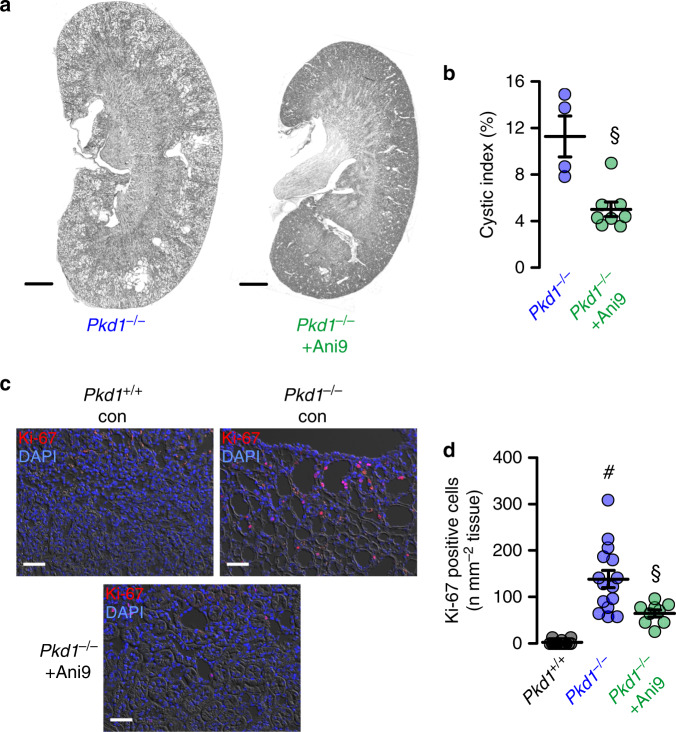
Ani9 inhibits polycystic kidney disease in a mouse model for ADPKD. **a** Representative images of kidneys from induced (*Pkd1*^*−/−*^) mice, untreated (daily i.p. injection of corn oil) (*n* = 8 kidneys analyzed from *n* = 4 animals) or treated with Ani9 in corn oil (Ani9, 0.5 mg/kg/day, i.p.) for 30 days (*n* = 8 kidneys analyzed from *n* = 4 animals). Scale bars 1000 µm. **b** Cystic index (%) 8 weeks after induction of *Pkd1*^*+/+*^ and *Pkd1*^*−/−*^ mice, untreated or treated with Ani9. Ani9 reduced cyst growth in *Pkd1*^*−/−*^ mice (^§^*P* = 0.001). ^§^Unpaired two-sided *t* test. **c**, **d** Proliferating cells identified in *Pkd1*^*+/+*^ and *Pkd1*^*−/−*^ kidneys by the proliferation marker ki-67 (red). Scale bars 50 µm. Proliferation was upregulated in *Pkd1*^*−/−*^ mice (^#^*P* < 0.0001), but was largely inhibited by Ani9 (^§^*P* = 0.021). ^#§^One-way ANOVA and Tukey’s post-hoc test. Number of ki-67 positive cells was normalized to the tissue area. Mean and error bars indicating ±SEM (25 independent images from *n* = 8 kidneys of *n* = 4 animals each). Mean and error bars indicating ±SEM. Source data are provided as a Source Data file.

**Fig. 9:**
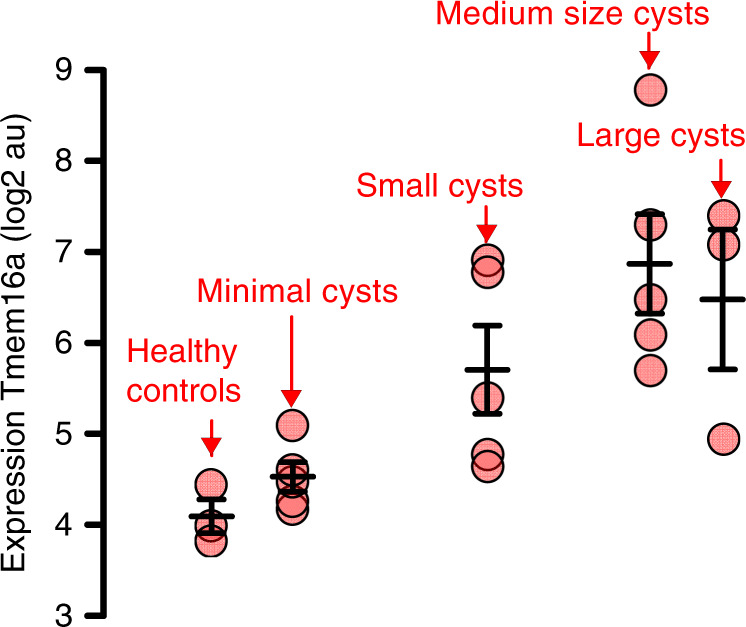
Expression of TMEM16A correlates with cyst size in ADPKD. Reanalysis of the public data set (GSE7869) from a global gene profiling study on renal cysts reported by Song et al.^31^. The data set comprises microarray expression data from cysts of different sizes from *n* = 5 *PKD1*-patients and from *n* = 3 kidneys of healthy patients. Reanalysis was performed using the Transcriptome Analysis Console (Thermo Fisher). Inspecting the probe covering *TMEM16A* (*ANO1*), a significant increase in *TMEM16A*-mRNA expression was found in the cystic tissue when compared to normal samples. Increase in *TMEM16A*-expression correlated with cyst size. Mean and error bars indicating ±SEM. Source data are provided as a Source Data file.

An even more pronounced inhibition of cyst growth is observed in animals treated with benzbromarone. Daily intraperitoneal injection of benzbromarone for 8 weeks significantly reduces renal cyst size when compared to sham treated animals (Fig. 7a, b). In line with these data, pathological weight gain caused trough polycystic kidneys is completely abolished by treatment with benzbromarone (Fig. 7c). Proliferative activity is analyzed using Ki-67. Knockdown of *Pkd1* largely enhances proliferation, which is almost completely eliminated by treatment with benzbromarone (Fig. 7d, e). Finally, we treat *PKd1*^*−/−*^ animals with Ani9, a recently discovered highly specific inhibitor of TMEM16A^29,30^. Ani9 likewise potently suppresses cyst formation in vivo (Fig. 8). Taken together, TMEM16A is a crucial protein and clinically relevant pharmacological target in ADPKD. The clinical relevance is further supported by re-analyzing an original data set from a global gene profiling study^31^, using the transcriptome analysis Console. When inspecting the probe for *Tmem16a*, a significant increase in *Tmem16a*-mRNA expression is found in the cystic tissue when compared to normal samples, and levels of expression correlated with cyst size (Fig. 9). These data compare well with a previous study that indicated an upregulation of TMEM16A in kidneys from ADPKD patients^8^. We therefore propose inhibitors of TMEM16A, such as niclosamide or benzbromarone, as therapeutics that potently suppress cyst growth in patients with ADPKD.

## Discussion

The present data demonstrate the crucial role of TMEM16A for the growth of renal cysts in a mouse model for ADPKD and in *Pkd1*-deficient plMDCK cells in vitro. TMEM16A was shown previously to be expressed at low levels in renal epithelial cells of normal human and mouse kidney, and was suggested to be upregulated in polycystic kidneys^8,19^. The present data now provide evidence that TMEM16A supports cyst growth by at least three mechanisms: (1) increasing intracellular Ca^2+^ signaling, (2) supporting Cl^−^ secretion, and (3) enhancing cell proliferation.

The essential role of CFTR for cAMP-regulated chloride secretion in intestine, airways, exocrine glands and numerous other epithelial organs is undisputed^5^. Traditionally, Ca^2+^ activated transport by TMEM16A is regarded as a separate and independent mechanism for transepithelial chloride secretion. Recent studies however suggest that both pathways overlap and cannot be separated easily. Thus CFTR-dependent secretion is also activated through Ca^2+^ dependent stimulation^32–34^. Knockout of TMEM16A in ciliated airway epithelial cells, intestinal epithelial cells and cell lines abrogated cAMP/CFTR-dependent chloride secretion^20^. In differentiated epithelial cells plasma membrane expression and activation of CFTR strongly depends on the presence of TMEM16A^7^. Plasma membrane tethering of the ER by TMEM16A and increase of compartmentalized Ca^2+^ signals close to the plasma membrane in conjunction with well described Ca^2+^ dependent modulations of CFTR function provides a mechanism for TMEM16A-dependent cyst formation^23,35^. Niclosamide, benzbromarone, and Ani9 attenuate compartmentalized Ca^2+^ signaling in renal epithelial cells and other cell types^30^.

The present report demonstrates enhanced Ca^2+^ signals in *Pkd1*^*−/−*^ cells that correlate with enhanced expression of TMEM16A (Figs. 4 and 5). Increase in purinergic Ca^2+^ signaling and increase in Ca^2+^ dependent chloride secretion upon knockout of *Pkd1* was observed in medullary (Figs. 4, 5 and Supplementary Fig. 6) as well as cortical tubular epithelial cells (Supplementary Figs. 7 and 8). Notably, deletion of *Pkd1* results in upregulation of hypoxia-inducible factor (HIF)-1α, which worsens cyst formation^17^. HIF1α augments expression of P2Y2R. Thus, expression of both P2Y2-receptor and TMEM16A are augmented in ADPKD^10^. Also, in the present study we demonstrate that in the absence of *Pkd1* (*Pkd1*^*−/−*^), expression of P2Y2 is augmented (Supplementary Fig. 2).

Enhanced Ca^2+^ mediated signaling in *Pkd1*^*−/−*^ cells is in conflict with previous studies, showing attenuated global Ca^2+^ levels in renal epithelial cells lacking expression of *PKD1*^36^. While these differences currently remain unexplained, the present results, showing enhanced basal intracellular Ca^2+^ concentrations in *Pkd1*^*−/−*^ cells, correspond to the enhanced basal short-circuit currents, basal Cl^−^ currents, and the basal halide permeability observed in *Pkd1*^*−/−*^ cells (Fig. 4, Supplementary Figs. 6 and 7). The present results are essentially identical to results previously reported for mouse M1 *Pkd1*^*−/−*^ cells^37^.

ER tethering by TMEM16A is of particular relevance for submembraneous Ca^2+^ signaling, i.e., Ca^2+^ store release and SOCE^22,23^. Conception et al. showed recently that store-operated Ca^2+^ entry regulates TMEM16A^38^. In fact TMEM16A-activity is controlled by intracellular Ca^2+^ as well as plasma membrane phosphatidylinositol 4,5-bisphosphate (PIP_2_)^30,39,40^. A large number of studies demonstrate the crucial role of TMEM16A for cell proliferation and cancer development (reviewed in refs. ^18,21^). Along this line, it was shown that knockdown of TMEM16A during kidney development affects ciliogenesis and reduces the number of renal glomeruli, thus causing albuminuria^41,42^.

The present data strongly suggest TMEM16A as a therapeutic target in ADPKD. Two well established FDA-approved drugs and potent inhibitors of TMEM16A, niclosamide and benzbromarone, strongly suppressed cyst growth in vivo (Figs. 5 and 6). Although both substances potently inhibit TMEM16A^12,13^, they are not specific to TMEM16A. Benzbromarone is primarily known as an uricosuric compound and niclosamide is used for the treatment of tapeworm infestations. Nevertheless, the present results suggest that the observed effects upon treatment with niclosamide, benzbromarone are due to inhibition of TMEM16A, which is further supported by the Ani9-mediated suppression of ADPKD. It has been reported earlier that degradation of TMEM16A is facilitated in the presence of the small molecule TMEM16A-inhibitor CaCCinhAO1^43^. Interestingly, we also found a remarkable reduction of TMEM16A-expression when cells were grown in the presence of niclosamide, Ani9, or benzbromarone (Supplementary Fig. 9). Thus, TMEM16A-inhibitors not only block Cl^−^ currents but also inhibit expression of TMEM16A upon long term treatment. Thus, genetic deletion of *Tmem16a* and inhibition by small molecules ultimately reduces the abundance of TMEM16A in the plasma membrane. This will lower ER-tethering and will attenuate local submembraneous Ca^2+^ signaling, with the functional consequences outlined here. We therefore propose inhibition of TMEM16-mediated intracellular signaling as a therapeutic principle in ADPKD.

## Methods

### Animals

Animal experiments were approved by the local institutional review board and all animal experiments complied with the with the United Kingdom Animals Act, 1986, and associated guidelines, EU Directive 2010/63/EU for animal experiments. Experiments were approved by the local Ethics Committee of the Government of Unterfranken/Wuerzburg (AZ: 55.2-2532-2-328). Mice with a floxed *PKD1* allele were generously provided by Prof. Dr. Dorien J.M. Peters (Department of Human Genetics, Leiden University Medical Center, Leiden, The Netherlands)^44^. Animals were hosted on a 12:12 h light:dark cycle under constant temperature (24 ± 1 °C) in standard cages. They were fed a standard diet with free access to tap water. Generation of mice with a tamoxifen inducible, kidney epithelium-specific *Pkd1*-deletion were described recently^17^. Mice carrying loxP-flanked conditional alleles of *Pkd1* were crossed with KSP-Cre mice in a C57BL/6 background (KspCreER^T2^;*Pkd1*^lox;lox^; abbreviated as *Pkd1*^−/−^). Mice carrying loxP-flanked alleles of *Tmem16a*^19^ were crossed to generate KspCreER^T2^;*Pkd1*^lox;lox^; *Tmem16a*^lox;lox^ double-knockout mice (abbreviated as *Pkd1*^−/−^/*T16a*^*−/−*^). Primers for genotyping are listed in Table 1. We used C57BL/6 single- and double-knockout males in the age of 8–10 weeks in the experiments.

**Table 1:** Primers used for genotyping.

Primer	Gene	Sequence 5′–3′
m*PKD1* loxPF	*Pkd1*	ACCCTTCCCTGAGCCTCCAC
m*PKD1* loxPR	*Pkd1*	CCACAGGGGAAGCCATCATA
F427 Va	*Ksp*	CATTCTCTCCCACTGAATGGA
F427 Vb	*Ksp*	ACAGAGTGGGGTTTGTGTCTG
inv	*Ksp*	AACTGTCCCCTTGTCATACCC
16aCKOf	*Tmem16a*	GGCTCTATCAATGTTCTGTTC
16aCKOr1	*Tmem16a*	CTCAAGTCCTCAAGTCCCAGTC

### Animal treatment

Conditional knockout was induced in *Pkd1*^−/−^ (*n* = 12) and *Pkd1*^−/−^/*T16a*^*−/−*^ (*n* = 12) mice by administrating tamoxifen (2 mg/kg body weight) dissolved in 5% ethanol and 95 % neutral oil, daily at postnatal days PN 20–22. Non-induced *Pkd1*^−/−^ (*n* = 9) served as controls. Induced *Pkd1*^−/−^ mice were fed with maintenance diet 1320 (Altromin, Lage, Germany) (*n* = 3) or with Altromin supplemented with 0.2% niclosamide (*n* = 3). All animals were sacrificed 10 weeks after induction with tamoxifen and kidneys were analyzed. Alternatively, *Pkd1*^−/−^ mice were maintained for 30 days and subsequently treated with benzbromarone (1 mg/kg/day) dissolved in corn oil or corn oil/DMSO (50 µl) by intraperitoneal injection for 30 days (*n* = 5). Mice were monitored twice a day by trained staff.

### Renal medullary and cortical primary cells

Mice were killed by cervical dislocation after exposure to CO_2_. Kidneys were removed and kept in ice-cold DMEM/F12 medium (Thermo Fisher Scientific, Darmstadt, Germany). The renal capsule was removed under germ-free conditions. Cortex and medulla were separated and chopped into smaller pieces of tissue using a sharp razor blade (Heinz Herenz, Hamburg, Germany). Tissues were incubated in Hanks balanced salt solution/DMEM/F12 (Life Technologies/Gibco^®^, Karlsruhe, Germany) containing 1 mg/ml collagenase type 2 (Worthington, Lakewood, USA) for 20 min at 37 °C. The digested tissue was passed through a 100 µm cell strainer (Merck KGaA, Darmstadt, Germany), transferred to a 50 ml falcon tube and washed with ice-cold PBS. After centrifugation at 5100 rpm for 4 min/4 °C, cells were resuspended. After resuspension, the cortical cell pellet was centrifuged at 17500 rpm for 30 min at 4 °C through a 45% Percoll (Ge Healthcare GmbH, Munich, Germany) 55% 2X PBS-Glucose gradient. After washing with ice-cold PBS, tubular preparations were maintained at 37 °C/5% CO_2_ in DMEM/F12 supplemented with 1% FBS, 1% Pen/Strep, 1% L-Glutamine (200 mM), 1% ITS (100×), 50 nM hydrocortisone, 5 nM triiodothyronine, and 5 nM Epidermal Growth Factor (Sigma Taufkirchen, Germany). After 24 h, primary cells grew out from isolated tubules.

### Principal-like MDCK cell cyst model and *PKD1*-knockout cells

Principal-like MDCK cells were grown at 37 °C, 21% O_2_ and 5% CO_2_ in modified MEM containing Earl’s balanced salt solution supplemented with 2 mM L-glutamine, 10% heat-inactivated FCS, 50 U/ml penicillin, and 50 μg/ml streptomycin. In vitro cyst assays were performed as described previously^8^. In brief cells were resuspended as a single-cell suspension in type I collagen and transferred to 24-well plates (3 wells per condition). Forskolin (FSK; 10 µM), ATP (10 µM), suramin (Sur; 100 µM), niclosamide ethanolamine (Niclo; 1 µM) and CFTRinh172 (CFTRi; 10 µM) were added to the medium at day 0, and medium was changed every 2 days. All substances were purchased from Sigma (Taufkirchen, Germany). After 5 days, images were taken from four random visual fields per well using Zeiss Primo Vert microscope and Zeiss Axiocam 105 camera (Zeiss Microscopy GmbH, Jena, Germany). Diameters of all captured spherical cysts were analyzed using ImageJ and spherical cyst volumes were calculated according to 4/3π*r*^3^.

A subclone of MDCK cells was used that resembles principal cells of the collecting duct as described earlier^9^. For genome editing pSpCas9(BB)-2A-puro-vectors (PX459) V2.0 (Addgene, Watertown, MA, USA) were used to generate a PKD^−/−^ cell line. The guide RNA was designed according to the algorithms provided by the Zhang laboratory (http://crispr.mit.edu/), which provided a quality score of 92. After ligation of the DNA-oligonucleotides with the vectors, 10^6^ cells were transfected using 10 µl polyethylenimine and 4 µg of the vector. After 24 h, cells were incubated with puromycine (3.5 µg/ml) for 48 h. Primers used for generation of the PKD1^−/−^ knockout cell line are shown in Table 2. Clones of cells were generated by dilution. For mutation screens, genomic DNA of single-cell clones was isolated and the CRISPR/Cas9 target region was amplified by PCR. Products were resolved by polyacrylamide gel electrophoresis. Genomic DNA of potential cell clones was amplified by PCR and cloned into pGL3 vector (Promega, Madison, WI, USA) and subjected to Sanger sequencing (eurofins, Nuernberg, Germany). The two main potential off-targets in DNA regions were tested for off-target effects by PCR amplification and polyacrylamide gel electrophoresis analysis. No off-target effects were detected.

**Table 2:** Primers used for generation of the MDCK-PKD1^−/−^ cell lines.

**Guide primers**	
Exon 1 fw	5′-CACCGTGCTCCGGGCATTGGACGTT
Exon 1 rev	5′-AAACAACGTCCAATGCCCGGAGCAC
**Cloning primers**	
Exon 1 cloning fw	5′-GCATGGTACCCAGAAAGGGAATGGCGCAG
Exon 1 cloning rev	5′-GCTAGCTAGCGCTTGGCTAATGACACCCAC
**Off-target primers**	
Chrom 34 fw	5′-ACGTAAGCGGCAAGAGTCAA
Chrom 34 rev	5′-CTGCCTTCATCACTCCCAGG
Chrom 3 fw	5‘-AATTGGCTGCTGTGCAAAGC
Chrom 3 rev	5′-CAGCCTCTCCCCATAGCAAG

### cAMP measurements

MDCK cells were grown in a pre-coated assay capture plate (cAMP-Screen Direct System P/N 4412186; Applied Biosystems, Foster City, CA, USA) in serum-free medium. Cells were exposed to serum-free control medium or medium supplemented with 10 µM forskolin. cAMP measurements were performed by the use of the GloMax-Multi Detection System (Promega) according to the manufacturer’s protocol.

### Western blotting

Protein was isolated from MDCK cells using a sample buffer containing 50 mM Tris-HCl, 150 mM NaCl, 10 mM EDTA, 1% Natriumdesoxychlorat, 0.1% SDS and 1% protease inhibitor mixture (Roche, cOmplete, EDTA-free, Mannheim, Germany) and 1% Triton X-100. Proteins were separated using an SDS polyacrylamide gel (10% for P2Y2R and 8% for TMEM16A) or NuPAGE 3–8% Tris-Acetate Protein Gels (Life Technologies/Gibco^®^, Karlsruhe, Germany) for detection of Polycystin-1 and CFTR. For detection of Polycystin1 and TMEM16A the proteins were blotted using an iBlot 2 Dry Blotting System (Thermo Fisher Scientific, Inc., Erlangen, Germany) on to a polyvinylidene difluoride membrane (GE Healthcare Europe GmbH, Munich, Germany). For detection of CFTR and P2Y2R, proteins were blotted using a Mini-PROTEAN TGX Stain-Free (Bio-Rad) using a semi-dry transfer unit (Bio-Rad) for 2–3 h. Membranes were then incubated with primary anti-CFTR (Alomone Labs; 1:500), anti-TMEM16A DOG-1 Polyclonal (Thermo Fisher; 1:1000) or anti-P2Y2R A20 (1:500, Santa Cruz Biotechnology) overnight. Proteins were visualized using horseradish peroxidase-conjugated secondary antibody and ECL detection. Beta-Actin was used as a loading control. Full uncropped blots are shown in Supplementary Figs. 10 and 11.

### Immunohistochemistry and antibodies

Two-micron thick transverse kidney sections were stained. For co-staining of CFTR and TMEM16A, anti-CFTR (rabbit; 1:100; Alomone, Jerusalem, Israel) and anti-TMEM16A (rabbit; 1:200, P80, described previously^8^) antibodies were used. As secondary antibodies, anti-rabbit IgG Alexa Fluor 555 and 488 antibodies (1:1000; Thermo Fisher Scientific, Inc., Erlangen, Germany) were used. Ki-67 staining was performed using a monoclonal anti-ki-67 antibody (rabbit; 1:100, Linaris, Dossenheim, Germany). Signals were amplified by the use of the Vectastain Elite ABC Kit (Vector Laboratories, Burlingame, CA) according to the manufacturer’s instructions. Signals were analyzed with a DM6000B fluorescence microscope (Leica, Wetzlar, Germany), and photographs were taken with a Leica DFC 450C camera.

Affinity purified polyclonal antiserum against mouse TMEM16A was produced in rabbits immunized with DPDAECKYGLYFRDGKRKVD (aa 44-63, N-terminus) or NHSPTTHPEAGDGSPVPSYE (aa 957-976, C-terminus), coupled to keyhole limpet hemocyanin (Davids Biotechnologie, Regensburg, Germany). Mouse kidneys were fixed by perfusion with 4% (v/v) paraformaldehyde and postfixed in 0.5 mol/L sucrose and 4% paraformaldehyde solution. Paraffin sections of 5 µm were blocked with 5% bovine serum albumin (BSA) and 0.04% Triton X-100 in PBS for 30 min. Cryo sections of 5 µm were incubated in 0.1% sodium dodecyl sulfate for 5 min and afterwards washed with PBS. Sections were incubated with primary antibodies (1:200) in 0.5% BSA and 0.04% Triton X-100 overnight at 4 °C. Additional primary antibodies were guinea pig anti-megalin (gift from Dr. F. Theilig, University of Freiburg, Germany), rabbit anti-CFTR (Alomone labs, # ACL-006), goat anti-AQP2 (Santa Cruz Biotechnology, Heidelberg, Germany), mouse anti-calbindin (Swant, Bellinzona, Switzerland), mouse anti-acetylated tubulin (Sigma-Aldrich, Munich, German), and rat anti-mouse Ki-67 (Dako Cytomation, Hamburg, Germany). After washing, sections were incubated with appropriate secondary antibodies for 1 h at room temperature (Alexa Fluor 488-labeled donkey anti-rabbit IgG (1:300, Invitrogen, Darmstadt, Germany), Alexa Fluor 546-labeled goat anti-mouse IgG (Molecular Probes), Alexa Fluor 546-labeled donkey anti-goat IgG (Molecular Probes), Cy5-labeled donkey anti-guinea pig IgG (Dianova), and Alexa Fluor 546-labeled goat anti-rat IgG (Molecular Probes). Sections were counterstained with Hoe33342 (1:200, Sigma-Aldrich). Coverslips were mounted with fluorescence mounting medium (Dako Cytomation, Hamburg, Germany). Immunofluorescence was detected using an Axiovert 200 microscope equipped with ApoTome and AxioVision (Zeiss, Germany).

### Immunohistochemistry and fluorescent signals

Ten random photographs were taken from the cortex of each kidney at a magnification of X200. Immunofluorescence (TMEM16A and CFTR) was analyzed as described previously^17^. Briefly, fluorescent signals were turned into 8-bit images after subtracting background (ImageJ) and a predefined threshold was used for all images to capture signals. Colocalization was visualized in white by use of ImageJ and an algorithm (http://rsb.info.nih.gov/ij/plugins/colocalization-finder.html, Laummonerie and Mutterer, Institut de Biologie Moleculaire des Plantes, Strasbourg, France). For quantification of ki-67, the color deconvolution algorithm (ImageJ) was applied to dissect the different signals, followed by binarization and particle analysis to obtain the ratio of the number of positive cells and cortex area (normalized to mm^2^ cortex tissue).

### Morphological analyses

Photographs from hematoxylin and eosin–stained kidney sections were taken at a magnification of ×25 and stitched to obtain single photographs of the whole transverse kidney sections using a Leica DM6000B microscope and a Leica DFC 450C camera. The whole kidney cortex was defined as region of interest using ImageJ and Cinteq 13HD creative pen display (Wacom, Düsseldorf, Germany). Next, we used an algorithm^17^ that separates normal tubule space from cystic area by defining diameters of noncystic tubules < 50 mm. The whole cortex cyst area was divided by the whole cortex area and defined as the cystic index.

### Ussing chamber

Medullary or cortical primary cells were grown as polarized monolayers on permeable supports (Millipore). Filters were mounted into a perfused micro Ussing chamber and the luminal and basolateral surfaces of the epithelium were perfused continuously with ringer solution (in mM: NaCl (145), KH_2_PO_4_ (0.4), K_2_HPO_4_ (1.6), Glucose (5), MgCl_2_ (1) Ca-gluconate (1.3)) at a rate of 6 ml/min (chamber volume 2 ml). All experiments were carried out at 37 °C under open-circuit conditions. Transepithelial resistance (Rte) was determined by applying short (1 s) current pulses (Δ*I* = 0.5 μA) and the corresponding changes in transepithelial voltage (Vte) were recorded continuously. Values for Vte were referred to the basolateral side of the epithelium. Rte was calculated according to Ohm’s law (Rte = ΔVte/ΔI). The equivalent short-circuit current (Isc) was calculated according to Ohm’s law from Vte and Rte (Isc = Vte/Rte).

### YFP-quenching assay

For YFP-quenching assays, primary renal cells were infected with lentiviral vectors to express halide-sensitive YFP_I152L_, as previously described^45^. Cells were isolated from 4 different mice and for each mouse 40 cells were measured. Quenching of the intracellular fluorescence generated by the iodide sensitive Enhanced Yellow Fluorescent Protein (EYFP-I152L) was used to measure anion conductance. YFP-I152L fluorescence was excited at 500 nm using a polychromatic illumination system for microscopic fluorescence measurement (Visitron Systems, Puchheim, Germany) and the emitted light measured at 535 ± 15 nm with a Coolsnap HQ CCD camera (Roper Scientific). Quenching of YFP-I152L fluorescence by I^−^ influx was induced by replacing 5 mM extracellular Cl^−^ with I^−^. Cells were grown on coverslips and mounted in a thermostatically controlled imaging chamber maintained at 37 °C. Cells were continuously perfused at 8 ml/min with Ringer solution and exposed to I^−^ concentration of 5 mM by replacing same amount of NaCl with equimolar NaI. Background fluorescence was subtracted, while auto-fluorescence was negligible. Changes in fluorescence induced by I^−^ are expressed as initial rates of maximal fluorescence decrease (Δ*F*/Δ*t*). For quantitative analysis, cells with low or excessively high fluorescence were discarded.

### Measurement of intracellular Ca^2+^ ([Ca^2+^]_*i*_)

Renal primary cells were loaded with 2 µM Fura-2/AM and 0.02% Pluronic F-127 (Invitrogen, Darmstadt, Germany)) in ringer solution (mmol/l: NaCl 145; KH_2_PO_4_ 0.4; K_2_HPO_4_ 1.6; Glucose 5; MgCl_2_ 1; Ca^2+^-Gluconat 1.3) for 1 h at room temperature. Fluorescence was detected in cells perfused with Ringer’s solution at 37 °C using an inverted microscope (Axiovert S100, Zeiss, Germany) and a high speed polychromator system (VisiChrome, Puchheim, Germany). Fura-2 was excited at 340/380 nm, and emission was recorded between 470 and 550 nm using a CoolSnap camera (CoolSnap HQ, Visitron). [Ca^2+^]_*i*_ was calculated from the 340/380 nm fluorescence ratio after background subtraction. The formula used to calculate [Ca^2+^]_*i*_ was [Ca^2+^]_*i*_ = *Kd* × (*R* − *R*_min_)/(*R*_max _− *R*) × (*S*_f2_/*S*_b2_), where *R* is the observed fluorescence ratio. The values *R*_max_ and *R*_min_ (maximum and minimum ratios) and the constant *S*_f2_/*S*_b2_ (fluorescence of free and Ca^2+^-bound Fura-2 at 380 nm) were calculated using 1 µmol/l ionomycin (Calbiochem), 5 µmol/l nigericin, 10 µmol/l monensin (Sigma), and 5 mmol/l EGTA to equilibrate intracellular and extracellular Ca^2+^ in intact Fura-2-loaded cells. The dissociation constant for the Fura-2•Ca^2+^ complex was taken as 224 nmol/l.

### Patch clamping

Patch-clamp experiments were performed in the fast whole-cell configuration. Patch pipettes had an input resistance of 2–4 MΩ, when filled with a cytosolic-like” pipette filling solution^46^, containing (mM) KCl 30, K-gluconate 95, NaH_2_PO_4_ 1.2, Na_2_HPO_4_ 4.8, EGTA 1, Ca-gluconate 0.758, MgCl_2_ 1.034, D-glucose 5, ATP 3. pH was 7.2, the Ca^2+^ activity was 0.1 µM. The extracellular bath perfusion was a Ringer solution containing (mmol/l) NaCl 145; KH_2_PO_4_ 0.4; K_2_HPO_4_ 1.6; Glucose 5; MgCl_2_ 1; Ca^2+^-Gluconat 1.3. The predicted equilibrium potential for Cl^−^ is around −39 mV. The membrane voltage as assessed in patch-clamp experiments was about −40 mV. The access conductance was measured continuously and was 30–140 nS. Currents (voltage clamp) and voltages (current clamp) were recorded using a patch-clamp amplifier (EPC 7, List Medical Electronics, Darmstadt, Germany), the LIH1600 interface and PULSE software (HEKA, Lambrecht, Germany) as well as Chart software (AD-Instruments, Spechbach, Germany). Data were stored continuously on a computer hard disc and were analyzed using PULSE software. In regular intervals, membrane voltages (*V*_c_) were clamped in steps of 20 mV from −100 to +100 mV relative to resting potential. Membrane conductance *G*_m_ was calculated from the measured current (*I*) and *V*_c_ values according to Ohm’s law.

### Materials and statistical analysis

All compounds used were of highest available grade of purity. Data are reported as mean ± SEM. Student’s *t* test for unpaired samples and ANOVA were used for statistical analysis. *P* < 0.05 was accepted as significant difference. Data are expressed as mean ± SEM. Differences among groups were analyzed using one-way ANOVA, followed by a Bonferroni test for multiple comparisons. An unpaired *t* test was applied to compare the differences between two groups. Wilcoxon signed-rank test for columns statistics was used for relative values. *P* < 0.05 was considered statistically significant.

### Reporting summary

Further information on research design is available in the Nature Research Reporting Summary linked to this article.

## Supplementary information

Supplementary Information

Reporting Summary

## Data Availability

All relevant data are available from the authors. A source data file has been included that contains all raw data underlying all reported averages in graphs and charts, as well as all uncropped versions of any gels or blots presented in the figures.

## Source data

Source Data
