# Supplementary material for: Cyst growth in ADPKD is prevented by pharmacological and genetic inhibition of TMEM16A in vivo
Source: Nat Commun. 2020 Aug 28;11:4320. doi: 10.1038/s41467-020-18104-5 (PMC7455562; doi:10.1038/s41467-020-18104-5)
Supplement: Supplementary file 1 — Supplementary Information [file 41467_2020_18104_MOESM1_ESM.pdf]

Cyst growth in ADPKD is prevented by pharmacological and genetic inhibition of TMEM16A *in vivo*

[1]Ines Cabrita[#], [2]Andre Kraus[#], [2]Julia Katharina Scholz, [2]Kathrin Skoczynski, [1]Rainer Schreiber[&], [1]Karl Kunzelmann[§,&], [2]Björn Buchholz[&]

Supplementary Information

Content:

- Supplementary Figure 1
- Supplementary Figure 2
- Supplementary Figure 3
- Supplementary Figure 4
- Supplementary Figure 5
- Supplementary Figure 6
- Supplementary Figure 7
- Supplementary Figure 8
- Supplementary Figure 9
- Supplementary Figure 10
- Supplementary Figure 11

Corresponding author
Karl Kunzelmann
e-mail: karl.kunzelmann@ur.de
Tel.: +49 (0)941 943 4302

Supplementary Figure 1

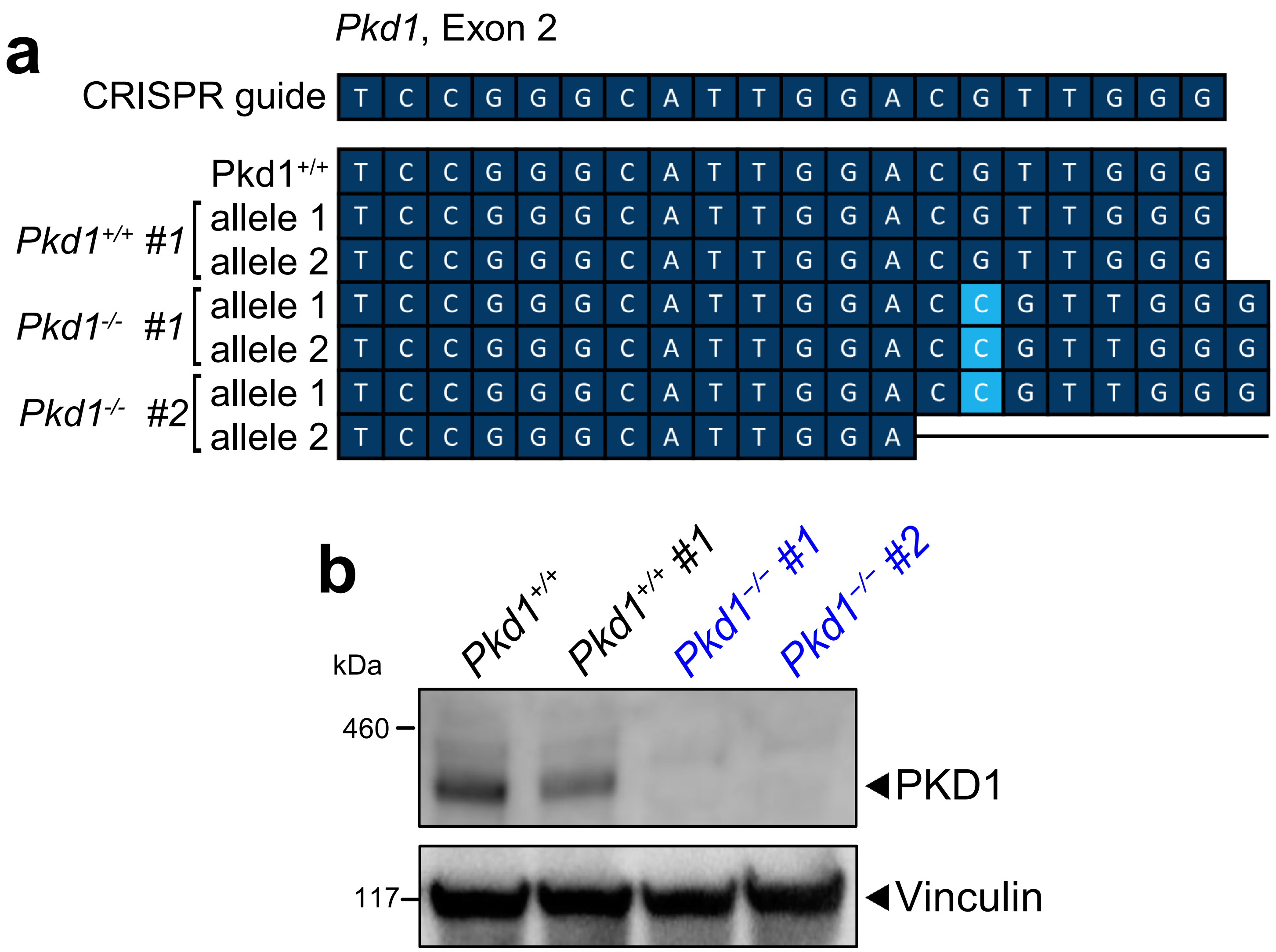

Supplementary Figure 1. Validation of the CRISPR/Cas9-mediated Pkd1 knockout in pIMDCK cells. a DNA sequences of CRISPR guide, wildtype (*Pkd1*$^{+/+}$), control clone (*Pkd1*$^{+/+}$ *#1*) and two *Pkd1* knockout clones (*Pkd1*$^{-/-}$ *#1 and #2*) confirming unaffected DNA sequences in wild type and control clone, and mutations in the two *Pkd1* knockout clones either resulting in insert mutations (light blue) or deletion (black line). **b** Representative Western blots for Polycystin-1 (PKD1, ~450 kDa) (vinculin as loading control, ~116 kDa) showing expression in wild type (*Pkd1*$^{+/+}$) and control clone (*Pkd1*$^{+/+}$ *#1*) and lack of Polycystin-1 expression in two *Pkd1* knockout clones (*Pkd1*$^{-/-}$ *#1 and #2*). (n=2 blots each). Source data are provided as a Source Data file.

Supplementary Figure 2

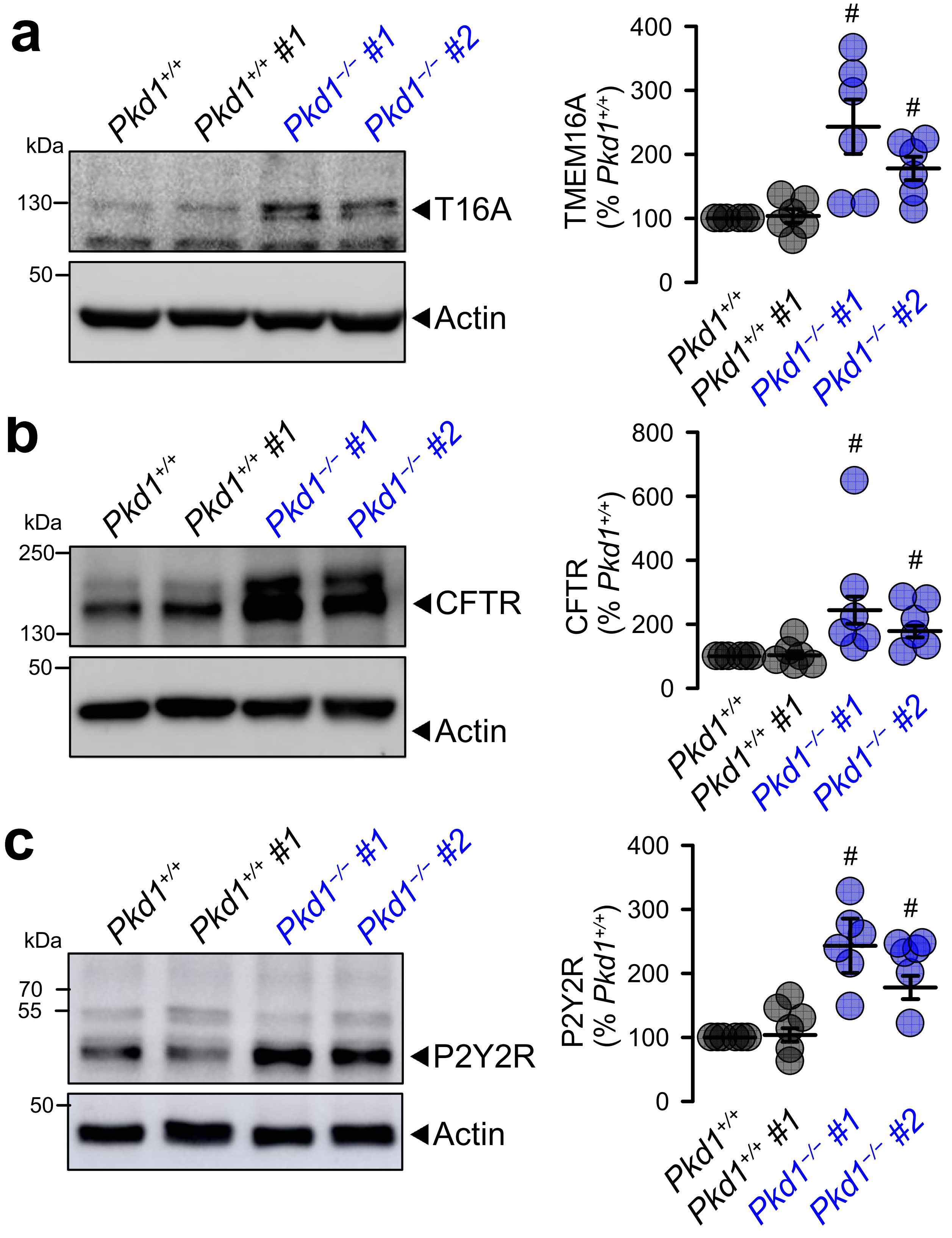

Supplementary Figure 2. PKD1-deficiency in pIMDCK cells leads to upregulation of TMEM16A, CFTR, and P2Y2R. a Western blotting for pIMDCK wildtype (*Pkd1*$^{+/+}$), control clone (*Pkd1*$^{+/+}$ *#1*) and Pkd1-deficient clones (*Pkd1*$^{-/-}$ *#1* and *Pkd1*$^{-/-}$ #2) detecting an increase of TMEM16A (~130 kDa) expression upon deletion of Pkd1 ($^{\#}$P=0.02 and $^{\#}$P=0.005) (n=6 replicates). **b** Western blotting from cell clones described in (a), detecting an increase of CFTR (~170 kDa) expression upon deletion of *Pkd1 in clone #1 and #2* ($^{\#}$P=0.02 and $^{\#}$P=0.007) (n=6 replicates). **c** Western blotting from cell clones described in (a) detecting an increase of P2Y2R (~47 kDa) expression upon deletion of *Pkd1* ($^{\#}$P=0.002 and $^{\#}$P=0.002) (n=6 replicates). Mean and error bars indicating ± SEM. §unpaired two-sided t-test. Source data are provided as a Source Data file.

Supplementary Figure 3

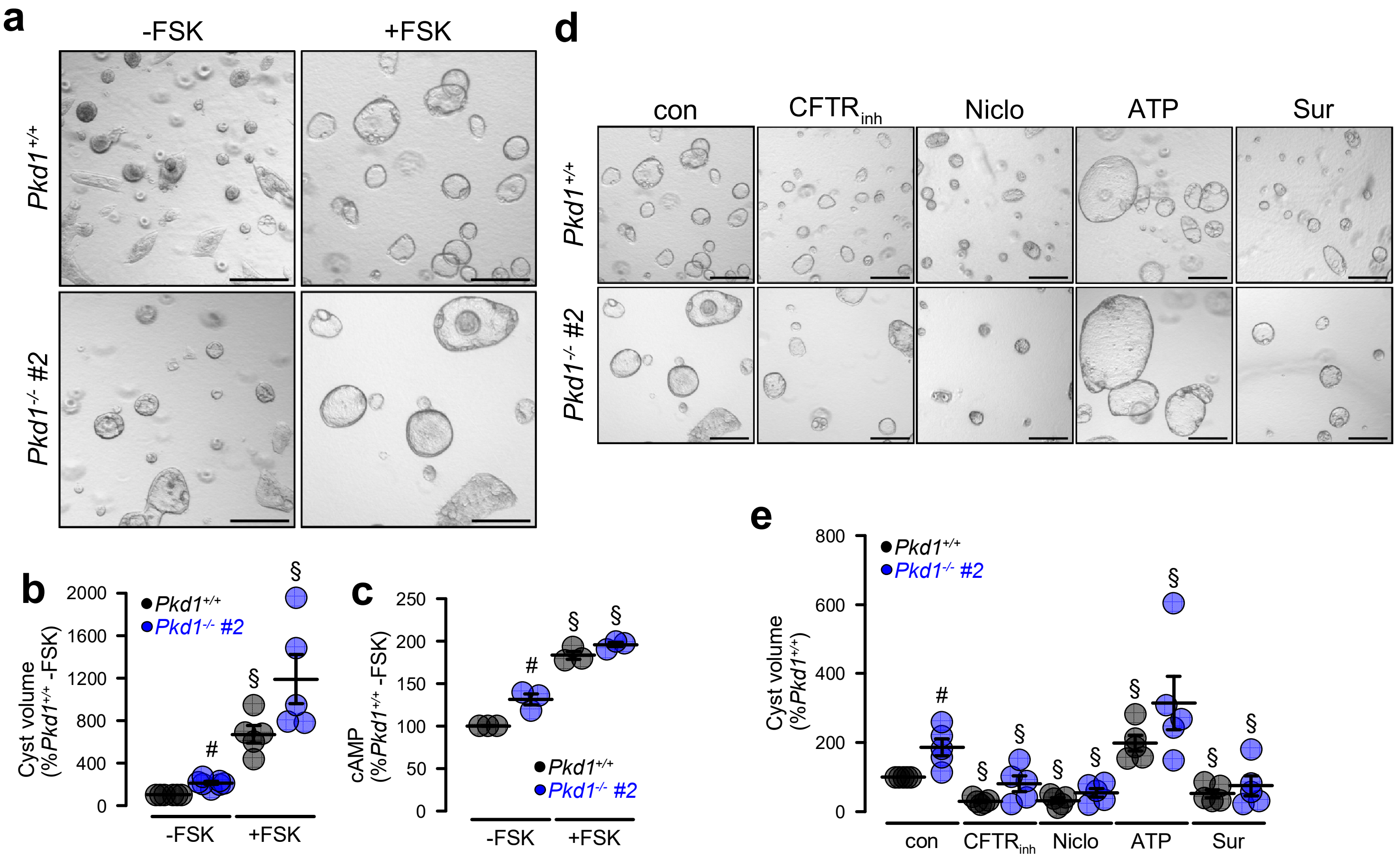

Supplementary Figure 3. Cyst formation by polycystin-1-deficient collecting duct cells depends on cAMP- and Ca^{2+}-activated Cl^- secretion. a,b. Wild type (*Pkd*$^{+/+}$) and polycystin-1-deficient (*Pkd1*$^{-/-}$) principal-like MDCK cells (clone #2) were cultured in a collagen matrix in the absence (-Fsk) or presence (+Fsk) of 10 µM forskolin. Forskolin induced cyst formation by *Pkd1*$^{+/+}$ cells within 5 days. *Pkd1*$^{-/-}$ cells demonstrated cyst formation even in the absence of FSK ($^{\#}$P=0.0001) and formed larger cysts in the presence of FSK (§P=0.002 and §P=0.012). (n=105 cysts examined in n=3 independent experiments). **c.** Deletion of polycystin-1 induced an increase in basal intracellular cAMP concentrations (-FSK) ($^{\#}$P=0.022). FSK-stimulation further enhanced cAMP levels in both *Pkd1*$^{+/+}$ and *Pkd1*$^{-/-}$ cells (§P=0.003 and §P=0.006) (each n=3 independent experiments). cAMP levels in *Pkd1*$^{+/+}$ cells were set to 100%. **d,e.** Enhanced ($^{\#}$P=0.026) cyst growth in *Pkd1*$^{-/-}$ and cyst growth in *Pkd1*$^{+/+}$ was strongly inhibited by CFTRinh172 (CFTRinh; 10 µM) (§P<0.0001 and §P=0.014), niclosamide (Niclo; 1 µM) (§P=0.0007 and §P=0.002), and suramin (Sur; 100 µM) (§P=0.014 and §P=0.019), but was further augmented by ATP (10 µM) (§P<0.014 and §P=0.04). (n=87 cysts examined in n=3 independent experiments). Bars 200 µm. Mean and error bars indicating ± SEM. $^{\#§}$unpaired two-sided t-test. Source data are provided as a Source Data file.

Supplementary Figure 4

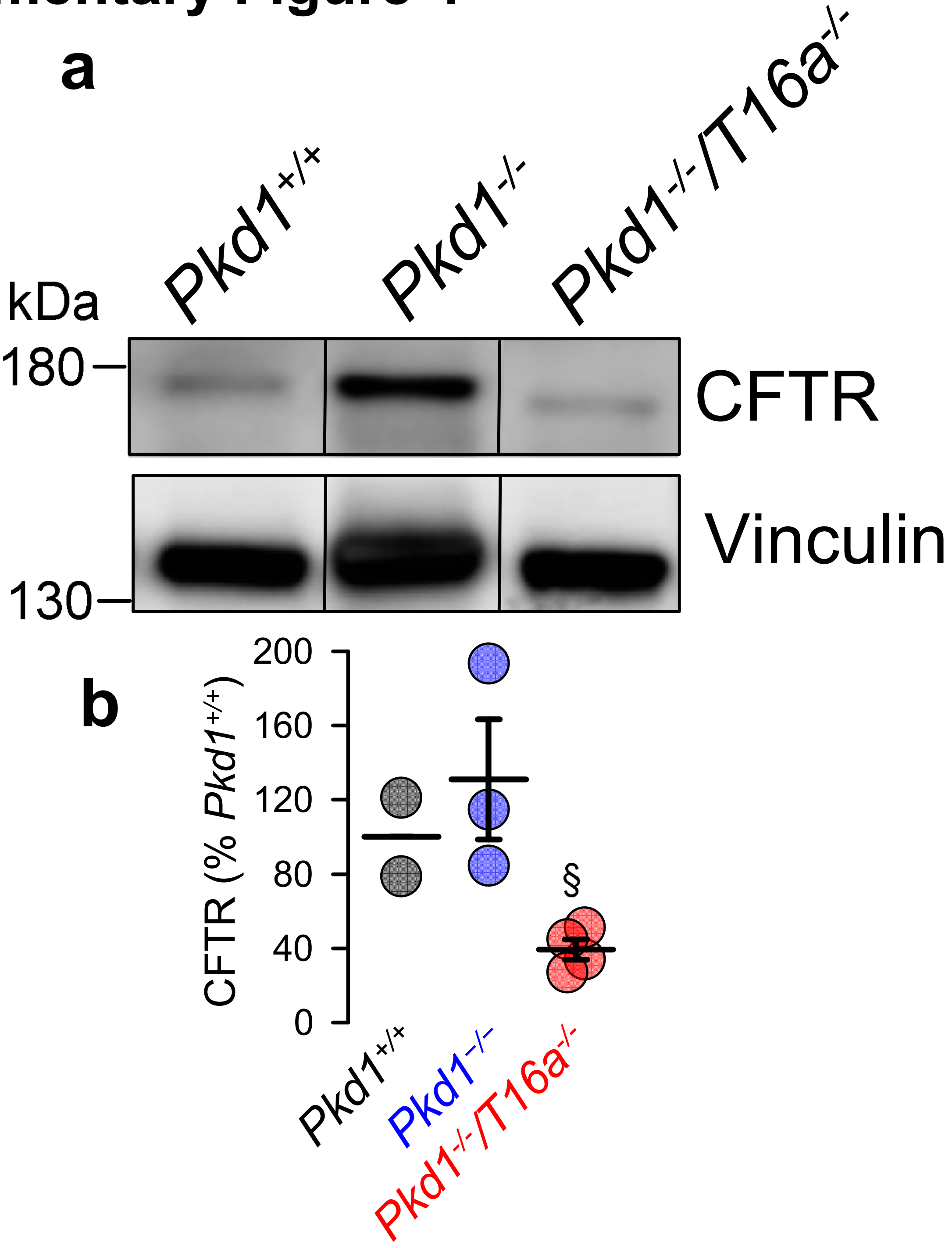

Supplementary Figure 4. Deletion of Tmem16a inhibits expression of CFTR. a,b. Analysis of CFTR (~ 170 kDa) expression in protein lysates from whole kidneys (Vinculin as loading control, ~ 116 kDa) 10 weeks after induction of *Pkd1*$^{-/-}$, and *Pkd1*$^{-/-}$/*Tmem16a*$^{-/-}$ double knockout mice. Densitometric quantification indicating non-significant upregulation of CFTR-expression in *Pkd1*$^{-/-}$ kidneys and reduced expression of CFTR in *Pkd1*$^{-/-}$/*T16a*$^{-/-}$ kidneys (§P=0.021). (n=3 independent blots). Mean and error bars indicating ± SEM. §unpaired two-sided t-test. Source data are provided as a Source Data file.

Supplementary Figure 5

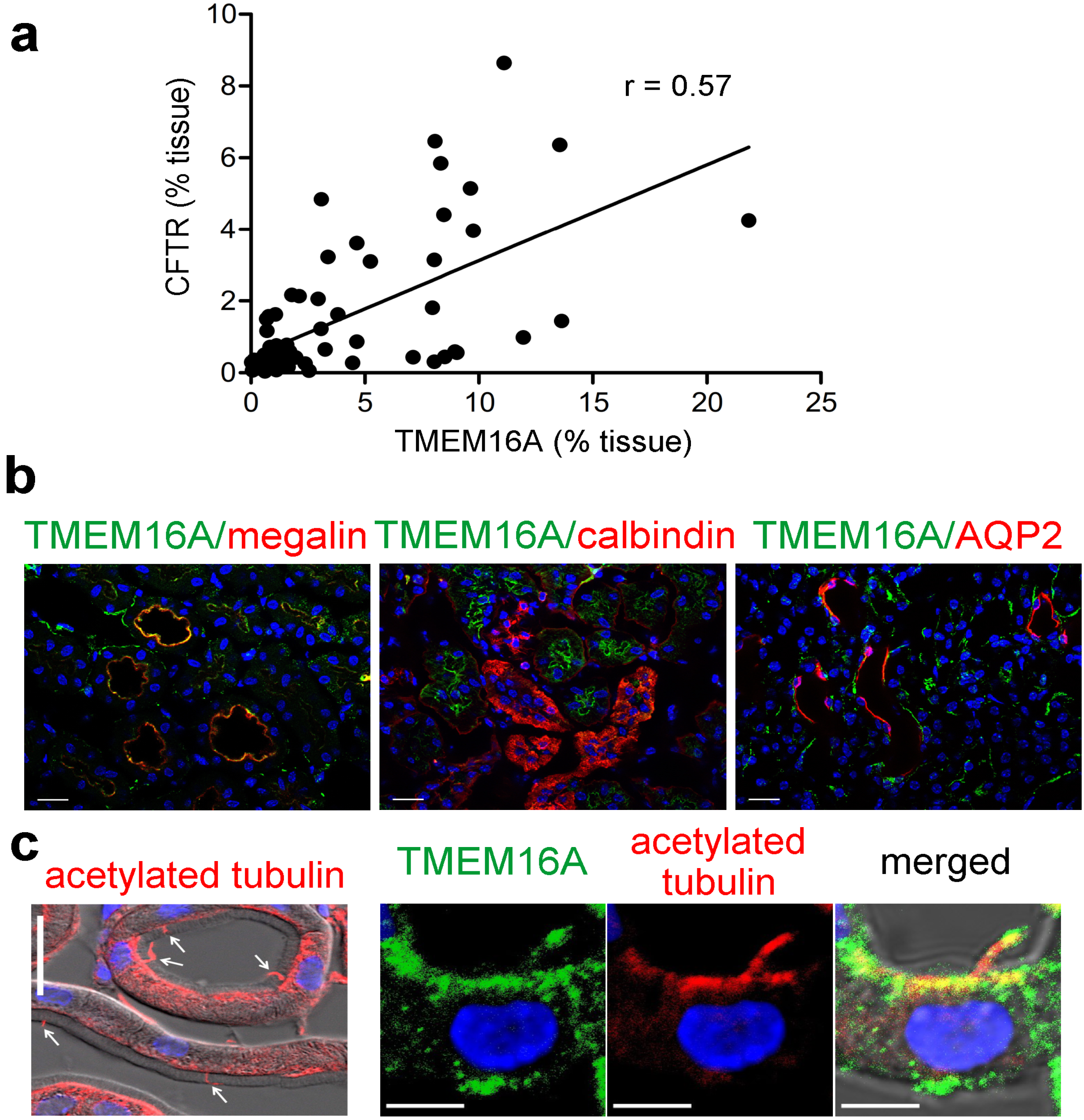

Supplementary Figure 5. Expression of TMEM16A and CFTR in murine kidney. a. Correlation between expression of TMEM16A and CFTR in the cyst epithelium of *Pkd1*$^{-/-}$ kidneys. Expression of TMEM16A and CFTR was normalized to tissue area. (n=25 images from n=5 mice). A correlation was found between expression of CFTR and TMEM16A (Pearson coefficient r=0.57). **b.** Expression of TMEM16A in *Pkd1*$^{+/+}$ kidneys. Colocalization was detected for TMEM16A and megalin (marker for proximal tubular epithelial cells), but not for TMEM16A and calbindin (marker for distal tubule) or TMEM16A and aquaporin 2 (AQP2) (marker for collecting duct). Representative images of kidneys from n=3 animals. Bar 20 µm. **c.** Primary cilia in *Pkd1*$^{+/+}$ renal tubules were identified using acetylated tubulin staining, which expressed TMEM16A. (Representative images of kidneys from n=3 animals). Bar 5 µm. Source data are provided as a Source Data file.

Supplementary Figure 6

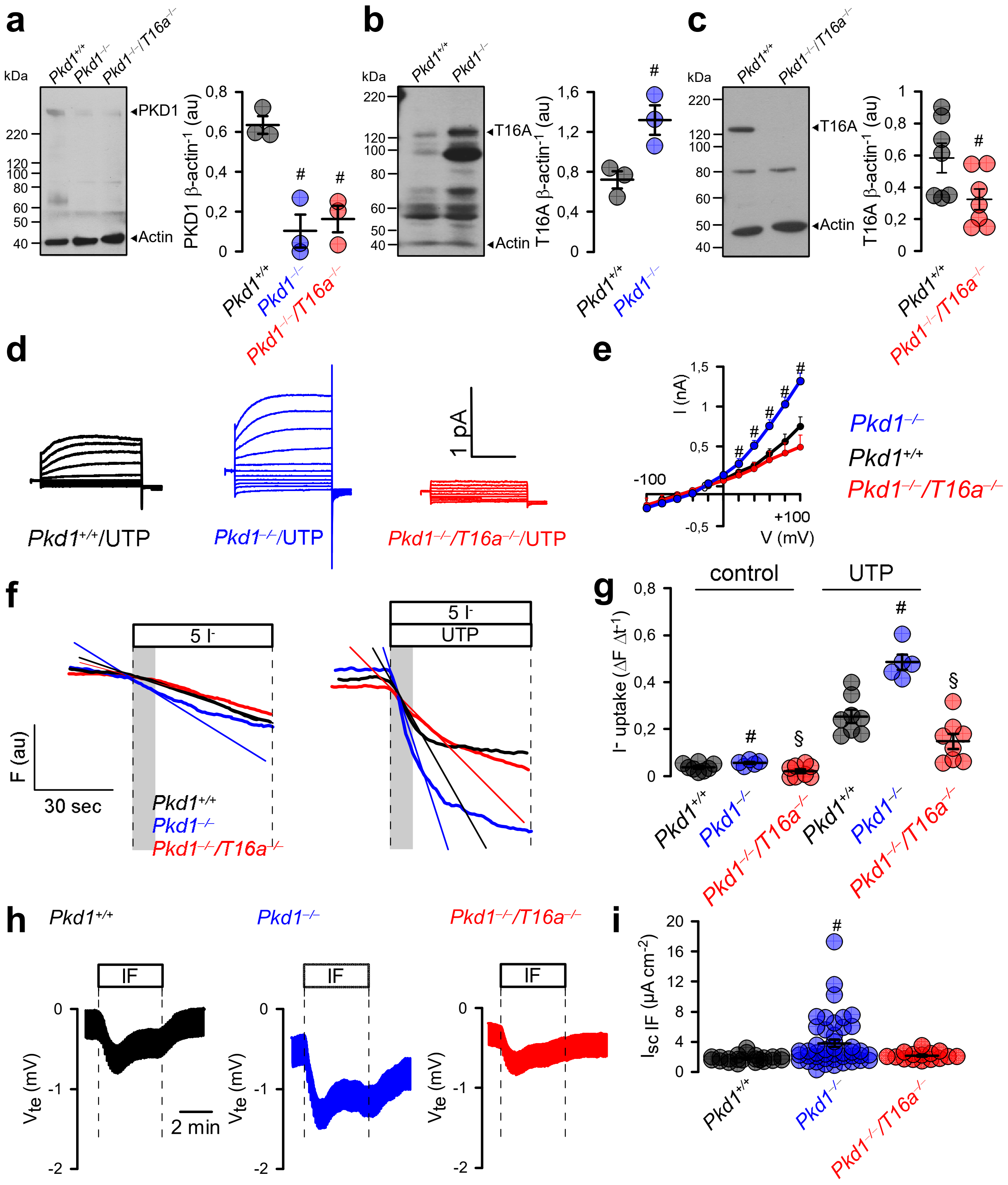

Supplementary Figure 6. TMEM16A is essential for upregulated chloride conductance in primary *Pkd1*$^{-/-}$ cells. a. Western blots and densitometric analysis indicating knockdown of Polycystin-1 (~ 450 kDa) in renal epithelial cells from *Pkd1*$^{-/-}$ ($^{\#}$P=0.003) and *Pkd1*$^{-/-}$*/T16a*$^{-/-}$ ($^{\#}$P=0.006) mice (n=67 cells from n=3 animals). $^{\#}$one-way ANOVA and Tukey's post-hoc test. **b.** Western blot and densitometric analysis indicating increased expression of TMEM16A (~ 130 kDa) in renal epithelial cells from *Pkd1*$^{-/-}$ mice ($^{\#}$P=0.025) mice (n=3 blots from n=3 animals). $^{\#}$unpaired two-sided t-test. **c.** Inhibited expression of TMEM16A in cells from *Pkd1*$^{-/-}$*/T16a*$^{-/-}$ mice ($^{\#}$P=0.025) (n=7 blots from n=3 animals). Actin was used as loading control. $^{\#}$unpaired two-sided t-test. **d,e.** Original recordings of whole cell currents and corresponding current/voltage relationships obtained in UTP-stimulated medullary primary epithelial cells isolated from *Pkd1*$^{+/+}$, *Pkd1*$^{-/-}$ and *Pkd1*$^{-/-}$*/T16a*$^{-/-}$ mice. ($^{\#}$P<0.02) (n=17 cells from n=3 animals each). $^{\#}$one-way ANOVA and Tukey's post-hoc test **f.** Basal and Ca^{2+}-activated (100 µM UTP) anion conductances detected by YFP quenching. Initial slopes correlate with size of anion conductance. **g.** Summary of initial slopes, (ΔFluorescence/s) indicating enhanced basal ($^{\#}$P=0.047) and UTP-activated ($^{\#}$P=0.0003) uptake in *Pkd1*$^{-/-}$ cells. Compared to *Pkd1*$^{-/-}$ cells, uptake was reduced in cells from *Pkd1*$^{-/-}$*/T16a*$^{-/-}$ mice (§P=0.015 and §P<0.0001). (n=126 cells from n=3 animals each). **h,i.** Original recordings and summaries of Ussing chamber experiments with primary medullary epithelial cells from *Pkd1*$^{+/+}$, *Pkd1*$^{-/-}$ and *Pkd1*$^{-/-}$*/T16a*$^{-/-}$ double knockout mice, showing enhanced Cl^- secretion by activation of CFTR with IBMX/forskolin (IF; 100 µM/ 2 µM) in *Pkd1*$^{-/-}$ cells ($^{\#}$P=0.007). (n=18 filters from n=3 animals each). $^{\#}$one-way ANOVA and Tukey's post-hoc test. Mean and error bars indicating ± SEM. Source data are provided as a Source Data file.

Supplementary Figure 7

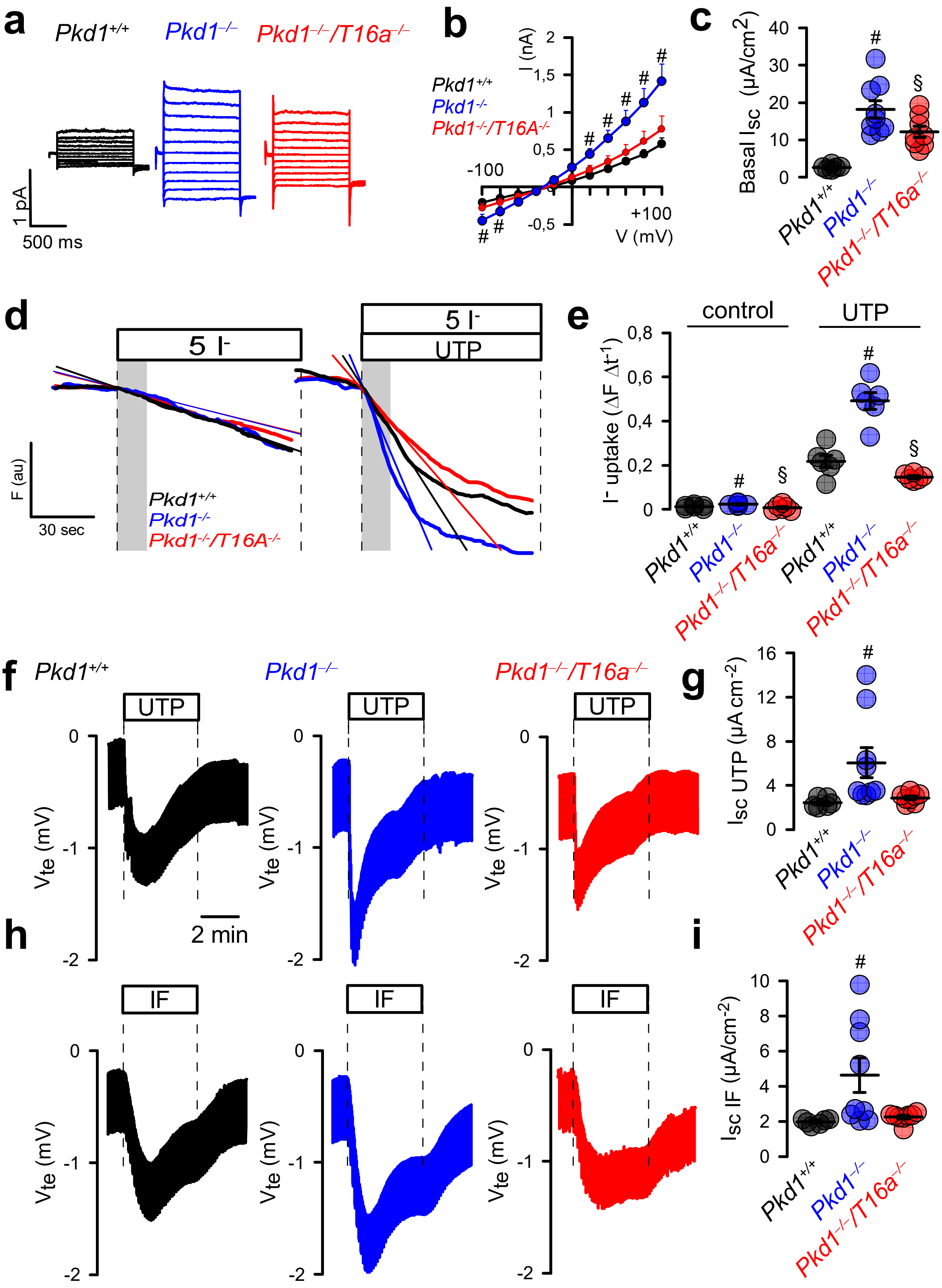

Supplementary Figure 7. TMEM16A is essential for upregulated Cl$^-$ conductance in cortical primary epithelial cells from *Pkd1$^{-/-}$* mice. a,b. Whole cell currents and corresponding current/voltage relationships obtained in primary cortical epithelial cells isolated from *Pkd1$^{+/+}$*, *Pkd1$^{-/-}$* and *Pkd1$^{-/-}$/T16a$^{-/-}$* mice. ($^{\#}$P<0.01) (n=19 cells from n=3 animals each). **c.** Summary of short circuit currents measured in Ussing chamber recordings showed upregulated transport in *Pkd1$^{-/-}$* cells ($^{\#}$P<0.0001), which was lower in *Pkd1$^{-/-}$/T16a$^{-/-}$* cells (§P=0.007). (n=15 filters from n=4 mice each). **d,e.** Basal and Ca^{2+}-activated (100 µM UTP) anion permeability assessed by YFP quenching. Summaries of YFP quenching (initial slope, ΔFluorescence/s) indicate upregulation of basal ($^{\#}$P<0.028) and UTP-activated ($^{\#}$P=0.0001) anion conductance in *Pkd1$^{-/-}$* cells, which was inhibited in *Pkd1$^{-/-}$/T16a$^{-/-}$* double knockout cells (§P<0.036 and §P=0.0002). (n=131 cells from n=4 animals each). **f-i.** Original Ussing chamber recordings and calculated short circuit currents indicate upregulated Cl$^-$ conductance activated by UTP (100 µM) ($^{\#}$P=0.039) (n=14 filters from n=3 animals each) or IBMX/Forskolin (I/F; 100 µM, 2 µM)

Supplementary Figure 8

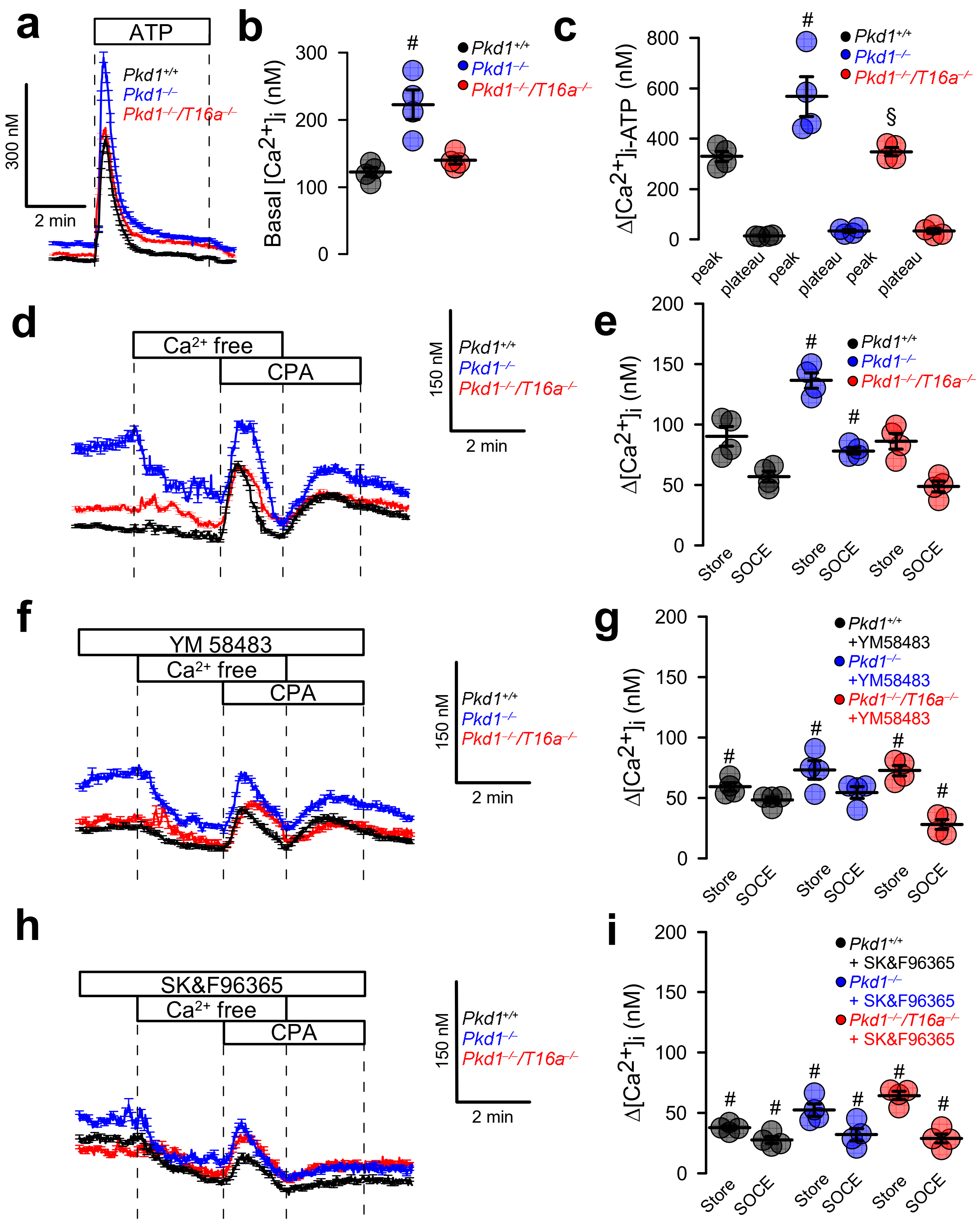

Supplementary Figure 8. Augmented Ca^{2+} signals in cortical primary epithelial cells. a-c. Original recordings and summaries for basal and ATP (100 µM) induced increase in intracellular Ca^{2+}. Ca^{2+} peak (ER store release) was enhanced in primary cells from *Pkd1*$^{-/-}$ ($^{\#}$P=0.001), but was reduced in cells from *Pkd1*$^{-/-}$*/T16a*$^{-/-}$ kidneys (§P=0.003). (n=137 cells from n=3 animals each). **d,e.** Original recordings and summaries for cyclopiazonic acid (CPA, 10 µM) induced store release and SOCE which were augmented in cells from *Pkd1*$^{-/-}$ ($^{\#}$P=0.002 and $^{\#}$P=0.01) (n=131 cells from n=3 animals each). **f-i.** Original recordings and summaries from experiments performed in the presence of Orai-inhibitor YM58483 and TRP-inhibitor SK&F96365 (both 5 µM). YM58483 inhibited store release in *Pkd1*$^{+/+}$ ($^{\#}$P=0.025), *Pkd1*$^{-/-}$ ($^{\#}$P<0.0001) *and Pkd1*$^{-/-}$*/T16a*$^{-/-}$ cells ($^{\#}$P=0.02) and SOCE in *Pkd1*$^{-/-}$*/T16a*$^{-/-}$ cells ($^{\#}$P=0.015)). (n=134 cells from n=3 animals each). SK&F96365 inhibited store release in *Pkd1*$^{+/+}$ ($^{\#}$P<0.0001), *Pkd1*$^{-/-}$ ($^{\#}$P<0.0001) *and Pkd1*$^{-/-}$*/T16a*$^{-/-}$ cells ($^{\#}$P<0.05), and SOCE in *Pkd1*$^{+/+}$ ($^{\#}$P=0.0007), *Pkd1*$^{-/-}$ ($^{\#}$P<0.0001) *and Pkd1*$^{-/-}$*/T16a*$^{-/-}$ cells ($^{\#}$P=0.024). (n=137 cells from n=3 animals each). Mean and error bars indicating ± SEM. $^{\#§}$one-way ANOVA and Tukey's post-hoc test. Source data are provided as a Source Data file.

Supplementary Figure 9

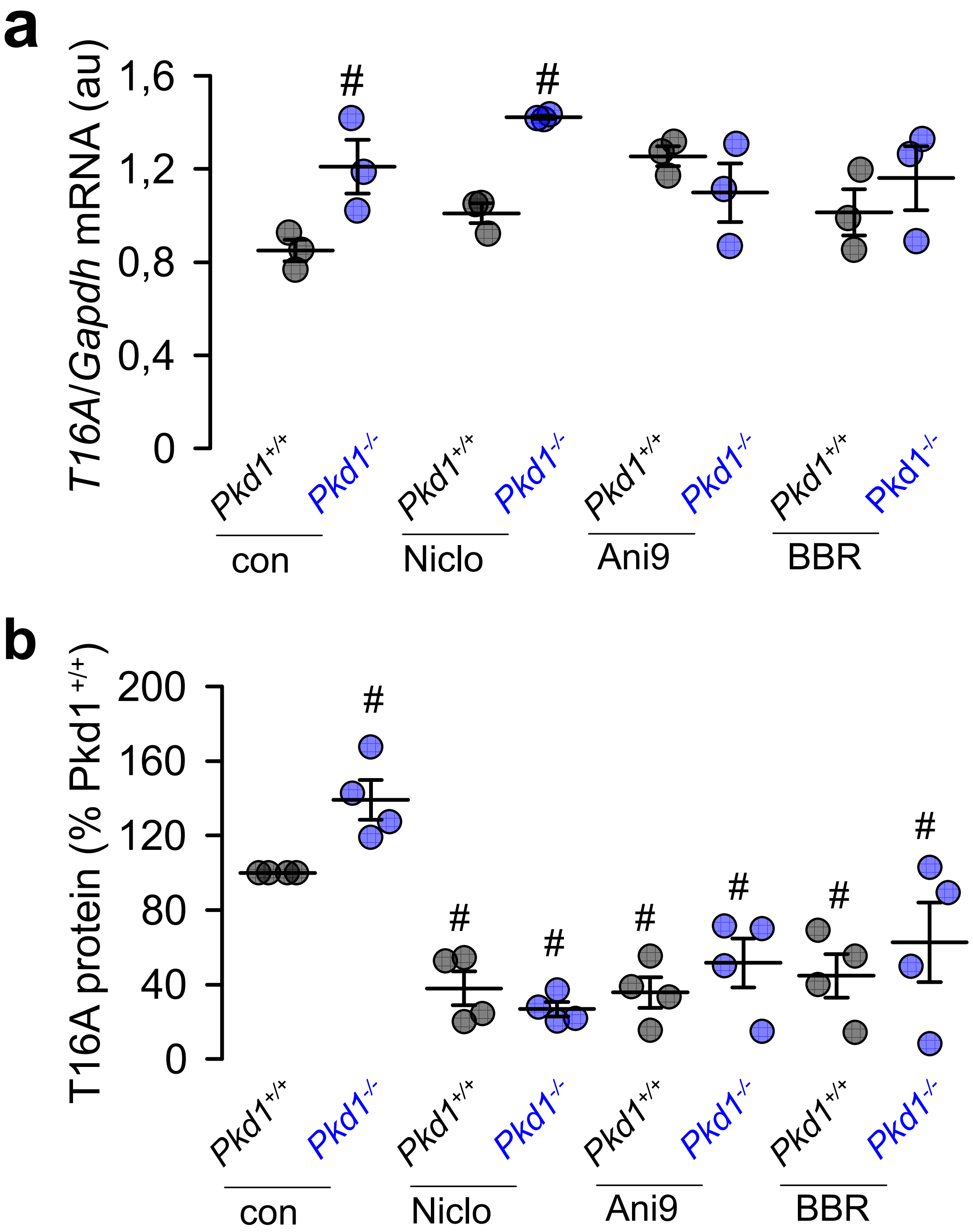

Supplementary Figure 9. TMEM16A-inhibitors block expression of TMEM16A in renal epithelial cells. a. Expression of TMEM16A mRNA assessed by semiquantitative RT-PCR and subsequent densitometric analysis in *Pkd1*$^{+/+}$ and *Pkd1*$^{-/-}$ renal epithelial cells. Expression was enhanced in *Pkd1*$^{-/-}$ cells under control ($^{\#}$P=0.04), and in the presence of Niclo ($^{\#}$P=0.005). (n=3 reactions for each inhibitor from n=2 animals). **b**. Expression of TMEM16A protein assessed by Western blotting and subsequent densitometric analysis in *PKD1*$^{+/+}$ and *PKD1*$^{-/-}$ renal epithelial cells. Expression was enhanced in *Pkd1*$^{-/-}$ cells under control ($^{\#}$P<0.035). Expression was inhibited in *Pkd1*$^{+/+}$ and *Pkd1*$^{-/-}$ cells by Niclo ($^{\#}$P=0.006 and $^{\#}$P<0.0001), Ani9 ($^{\#}$P=0.004 and $^{\#}$P=0.002), and BBR ($^{\#}$P=0.018 and $^{\#}$P=0.018) (n=4 blots for each inhibitor from n=2 animals). Cells were grown in the absence or presence of the TMEM16A inhibitors niclosamide (Niclo; 0.5 µM), Ani9 (1 µM) and benzbromarone (BBR; 5 µM) for 72h. Mean and error bars indicating ± SEM. $^{\#}$unpaired two-sided t-test. Source data are provided as a Source Data file.

Supplementary Figure 10

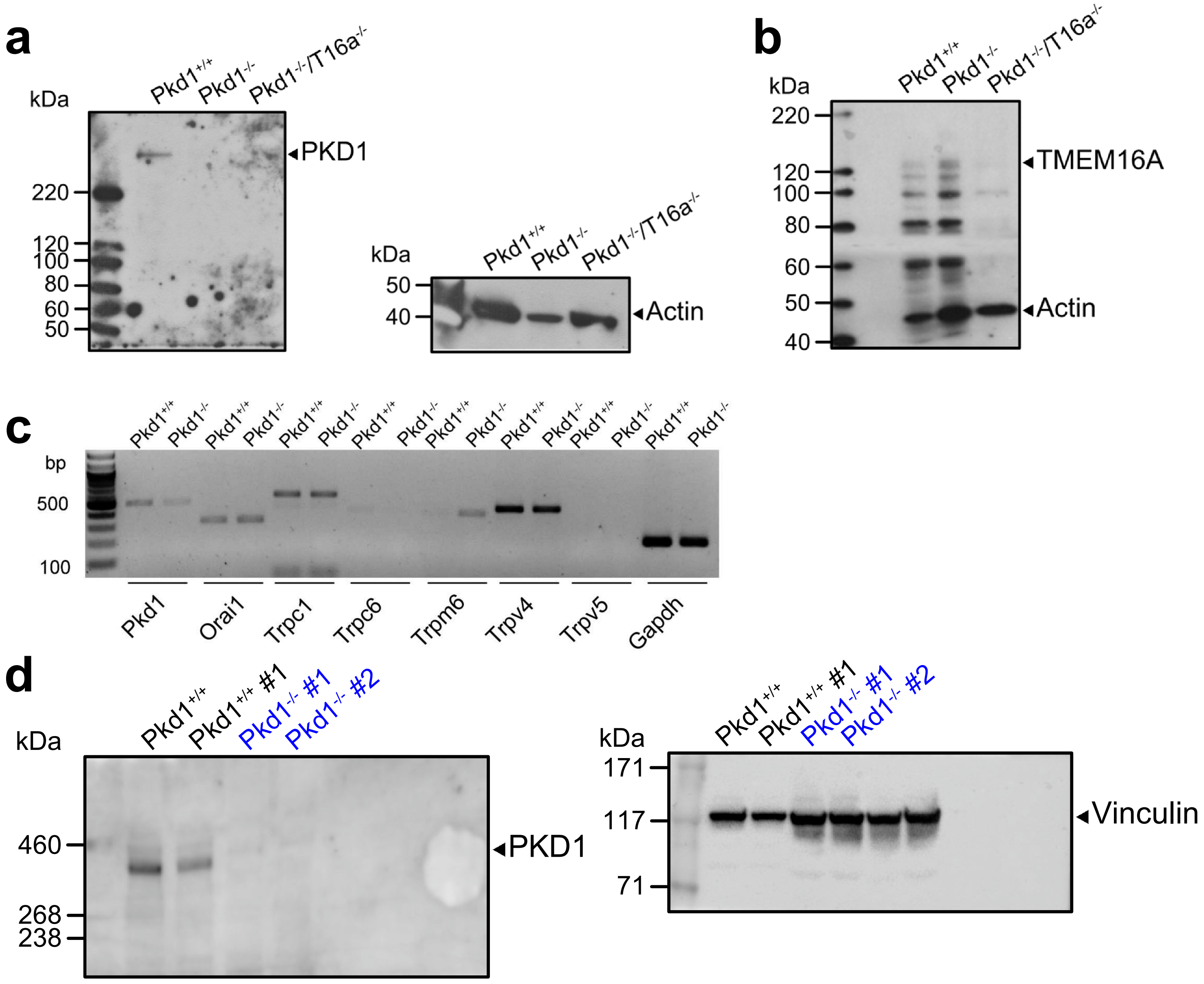

Supplementary Fig. 10. Original uncropped blots and gels. Blots shown belong to **a** Fig. 2a, **b** Fig. 2c, and **c** Fig. 5e, **d** Supplementary Fig. 1b. Molecular weight markers (kDa or bp) are shown at the left side of the blots.

Supplementary Figure 11

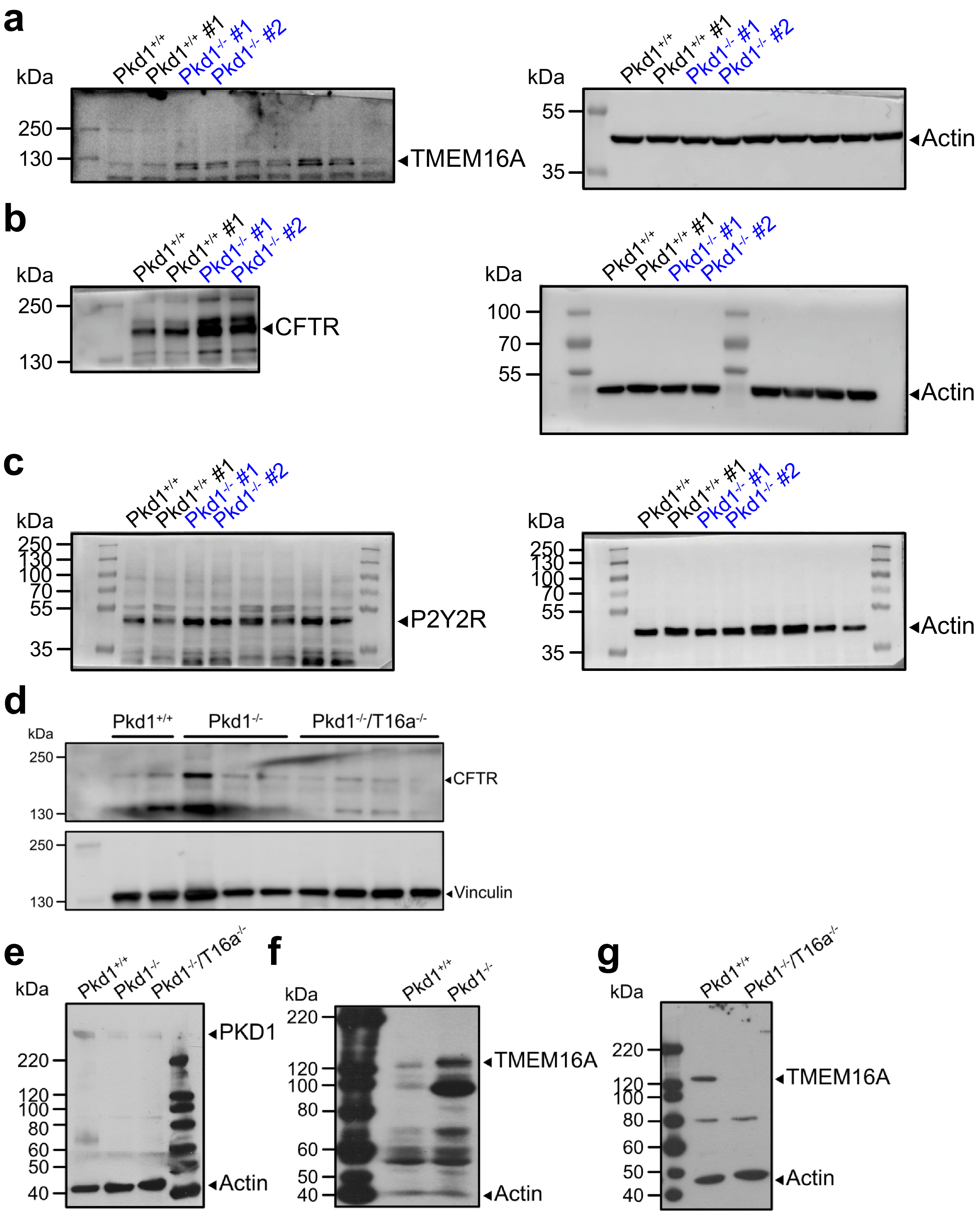

Supplementary Fig. 11. Original uncropped blots. Blots shown belong to **a-c** Supplementary Fig. 2a-c, **d** Supplementary Fig. 4a, **e-g** Supplementary Fig. 6a-c. Molecular weight markers (kDa or bp) are shown at the left side of the blots.
